# Multiplex immunohistochemistry accurately defines the immune context of metastatic melanoma

**DOI:** 10.1038/s41598-018-28944-3

**Published:** 2018-07-24

**Authors:** H. Halse, A. J. Colebatch, P. Petrone, M. A. Henderson, J. K. Mills, H. Snow, J. A. Westwood, S. Sandhu, J. M. Raleigh, A. Behren, J. Cebon, P. K. Darcy, M. H. Kershaw, G. A. McArthur, D. E. Gyorki, P. J. Neeson

**Affiliations:** 1 Cancer Immunology Research, Peter MacCallum Cancer Centre, 305 Grattan Street, Melbourne, 3000 Australia; 2 Division of Cancer Medicine Melanoma Program, Peter MacCallum Cancer Centre, 305 Grattan Street, Melbourne, 3000 Australia; 3 Division of Cancer Surgery, Peter MacCallum Cancer Centre, 305 Grattan Street, Melbourne, 3000 Australia; 4 Sir Peter MacCallum Department of Oncology, The University of Melbourne, Parkville, Victoria 3052 Australia; 5 Olivia Newton John Cancer Research Institute, Heidelberg, Victoria 3084 Australia; 6 Department of Surgery, University of Melbourne, Parkville, Victoria 3052 Australia; 7 School of Cancer Medicine, La Trobe University, Bundoora, 3086 Australia

## Abstract

A prospective study explored the heterogeneous nature of metastatic melanoma using Multiplex immunohistochemistry (IHC) and flow cytometry (FACS). Multiplex IHC data quantitated immune subset number present intra-tumoral (IT) vs the tumor stroma, plus distance of immune subsets from the tumor margin (TM). In addition, mIHC showed a close association between the presence of IT CD8^+^ T cells and PDL1 expression in melanoma, which was more prevalent on macrophages than on melanoma cells. In contrast, FACS provided more detailed information regarding the T cell subset differentiation, their activation status and expression of immune checkpoint molecules. Interestingly, mIHC detected significantly higher Treg numbers than FACS and showed preferential CD4^+^ T cell distribution in the tumor stroma. Based on the mIHC and FACS data, we provide a model which defines metastatic melanoma immune context into four categories using the presence or absence of PDL1^+^ melanoma cells and/or macrophages, and their location within the tumor or on the periphery, combined with the presence or absence of IT CD8^+^ T cells. This model interprets melanoma immune context as a spectrum of tumor escape from immune control, and provides a snapshot upon which interpretation of checkpoint blockade inhibitor (CBI) therapy responses can be built.

## Introduction

Increased tumor infiltrating lymphocytes (TILs) correlate with better outcome in many human cancers^1–6^ and were originally defined by pathologists on hematoxylin and eosin (H&E) sections, where TIL location and number was a key prognostic indicator in melanoma^7–10^. The term TIL also described lymphocytes harvested from melanoma biopsies^11^, analyzed by FACS, and assessed for anti-tumor responses (cytotoxicity and cytokine secretion). In addition, TILs describes T cells derived from the tumors of patients with metastatic melanoma that were expanded *ex vivo* and then re-infused, following lymphodepletion, as a successful form of adoptive immunotherapy^12^. Thus, over a 35 year period, the term ‘TIL’ has evolved into three distinct concepts. Whilst all of these have critical clinical importance, the flexible use of the term TIL created confused semantics around what truly defines a TIL. To clarify this issue we compared the immune context of melanoma patient biopsies by both FACS and multiplex IHC. Multiplex IHC is a powerful investigative tool which provides objective quantitative data describing the tumor immune context in both immune subset number and location^13^. To do this, the OPAL staining panel contains monoclonal antibodies directed to specific markers, which together define the immune subsets present. In addition, a tumor marker (eg SOX-10) is included to define the melanoma cells in the tumor. Following imaging, the precise x-y co-ordinate of every cell in the tissue section can be resolved to reveal whether individual immune subset cells are located within the tumor (ie a true TIL) or within the tumor stroma (a tumor associated lymphocyte). Thus, mIHC provides accurate immune context information describing the heterogeneity of ‘T cell inflamed’ versus immune excluded tumors. In contrast, FACS assessment of melanoma TILs provides a detailed description of T cell subsets, their differentiation and immune checkpoint expression. However, FACS analysis is performed on a cell suspension so histological location is lost. In this study, we compare TIL data derived from tissue sections (via mIHC) to TIL derived from a cell suspension (via FACS). We also explore how both sets of TIL data can be used to better inform the immune context of patient tumors for therapeutic decisions.

## Results

Tumor tissue from 21 patients was used for this study (Supplementary Table 1). Patients had a median age of 70 years and underwent surgery for stage III (38%) or stage IV (62%) disease. Most specimens were cutaneous/subcutaneous (48%) or nodal (33%). Most patients were treatment naïve with only 21% having received previous immunotherapy. The entire cohort had tissue evaluable by flow cytometry (Supplementary Table 2) however only 19 patients had tissue evaluable by mIHC (Supplementary Table 3).

### Multiplex IHC is a powerful investigative tool and can be used to assess the immune context of metastatic melanoma

We used H&E and OPAL-stained FFPE sections to describe the immune context of melanoma from multiple metastatic sites; example H&E and mIHC images are shown of melanoma resected from subcutaneous (Supplementary Fig. 1), lymph nodes (Supplementary Fig. 2) and visceral organs (Supplementary Fig. 3). The H&E sections were examined by a pathologist and regions where TILs were present (‘T cell inflamed’ or hotspots) identified. In addition, regions of melanoma with ‘immune exclusion’ were also revealed. The entire melanoma section was imaged on the Vectra system under low magnification to reveal an overarching immune context including assessment of TIL density and distribution. Select high powered fields (HPF) were imaged to reveal details of the immune context with resolution sufficient to describe immune subsets and precise tissue location of individual cells. Composite images were analyzed using inForm® software to define cells as either melanoma (SOX10^+^) or T cells subsets (CD3^+^CD4^+^, CD3^+^CD8^+^, CD3^+^CD4^+^FoxP3^+^, CD3^+^ CD4^−^CD8^−^). Using the tissue segmentation function, the tumor region was defined by SOX10^+^ melanoma cells, and the tumor stroma region comprised SOX10^−^ cells (Supplementary Fig. 4). Representative images of melanoma metastasis from the same tissue site showed immune context heterogeneity in melanoma metastases between patients (Supplementary Figs 1–3). For example, a subcutaneous metastasis resected from patient MelTIL006 (Supplementary Fig. 1) showed moderate TIL distribution and density. T cells were present in the tumor stroma as well as penetrating the tumor. In addition, this tumor expressed PD-L1 at high levels throughout. By contrast, a subcutaneous metastasis resected from patient MelTIL024 (Supplementary Fig. 1) showed a low TIL distribution and density, with no PDL1 expression.

**Figure 1:**
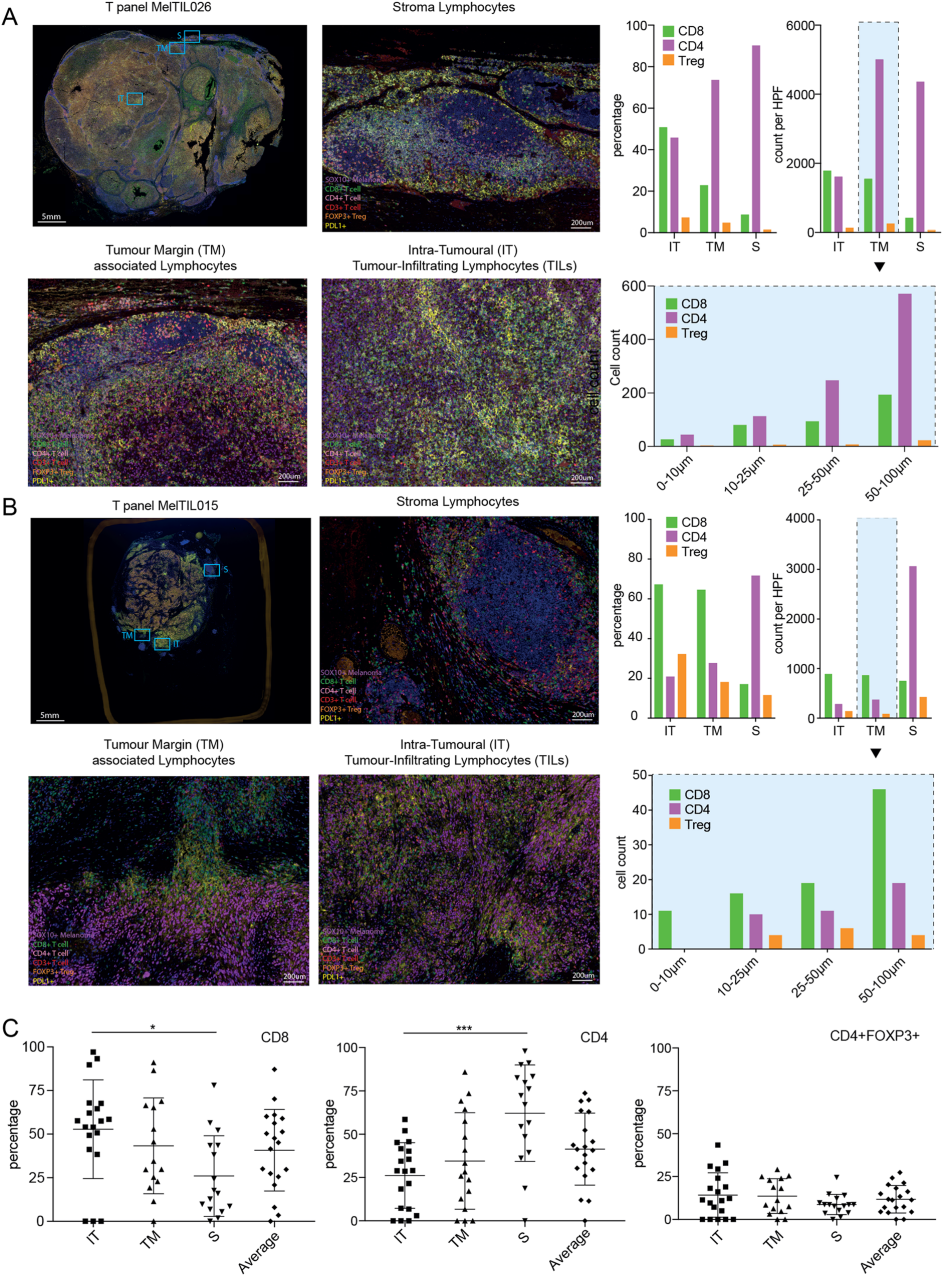
Multiplex IHC (mIHC) defines the precise location of immune subsets in metastatic melanoma. FFPE sections of melanoma were stained by OPAL “T-cell Panel” for SOX10, PDL1, CD3 (red), CD8, CD4 and FOXP3. Stained sections were imaged on the Vectra Automated Imaging System and composite images displayed for Mel TIL026 ((**A**) pelvic lymph node) and MelTIL015 ((**B**) visceral). Using the low power image, high-powered Fields (HPFs) were selected from specific tumour regions including intra-tumoral (IT), tumour margin (TM) and stromal regions (S). Data displayed for the T cell panel shows the distribution of tumour cells (purple), PDL1 (membranous yellow) on either melanoma or infiltrating APCs, CD8+ T cells (green), CD4+ T cells (pink) and T regulatory T cells (orange nucleus). Cell segmentation and phenotyping (Supplementary Fig. 1) enabled quantitation expressed as counts and percentage of total cells per high-powered field (HPF) for different T cell subsets (**A**,**B**). Cell distance measurements (Supplementary Fig. 2) enabled detailed distance analysis of T cell subsets from the tumour margin (boxed graph). In (**C**) collated data for all Mel-TIL samples in the study cohort is depicted, including the % of T cell subsets (CD8^+^, CD4^+^ and Tregs) in three tumour regions (IT, TM and S) as well as the average of these regions. Statistical analysis (Mann Whitney) was performed across the cohort, significantly different data is represented by *(p < 0.05) and ***(p < 0.005).

Multiplex IHC also accurately described differences in the immune context across multiple regions of metastatic melanoma. Example high power fields of the T cell panel (CD8^+^ T cells, CD4^+^ T cells and Treg) are shown for the tumor margin (TM), intra-tumor (IT) and peri-tumoral (stroma) regions in patients MelTIL026 and MelTIL015 (Fig. 1). These representative data showed distinct differences in T cell subsets between regions of the same tumor. For MelTIL026 (Fig. 1A), the majority of T cells were CD4^+^, prominent in the TM and stroma regions, whereas lower numbers of CD4^+^ and CD8^+^ T cells were present in the IT region. For MelTIL015 (Fig. 1B), T cell numbers were reduced compared to that observed in MelTIL026; CD4^+^ T cells were prominent in the stroma region and CD8^+^ T cells were lower in number and equally spread across IT, TM and stroma regions. Interestingly, mIHC data for immune subsets (counts per HPF and %) can reveal differences in the immune context within tumors as well as between tumors. Whilst T subset counts (counts per HPF) and % were equivalent in each region for MelTIL026 (brisk immune infiltrate), this was not the case for MelTIL015 (patchy immune infiltrate).

To explore the stromal distribution of T cell subsets further, we examined the cell counts for each subset in increasing distance from the tumor margin for MelTIL026 (Fig. 1A) and MelTIL015 (Fig. 1B). This revealed that CD4^+^ T cells increased in number from the TM up to 100 μm into the stroma for MelTIL026 (Fig. 1A). In contrast, MelTIL015 (Fig. 1B) showed increasing CD8^+^ (but not CD4^+^) T cells from the TM up to 100 μm into the stroma suggesting that the majority of stromal CD4^+^ T cells in MelTIL015 were greater than 100 μm from the TM. Collated mIHC data for all metastatic melanoma specimens shows T cell subset distribution (intra-tumoral, tumor margin and stroma) (Fig. 1C). We observed a significantly higher percentage of IT vs stromal CD8^+^ T cells (p < 0.05), and an inverse relationship for CD4^+^ T cells, with a higher percentage in the stroma than IT (p < 0.005).

**Figure 2:**
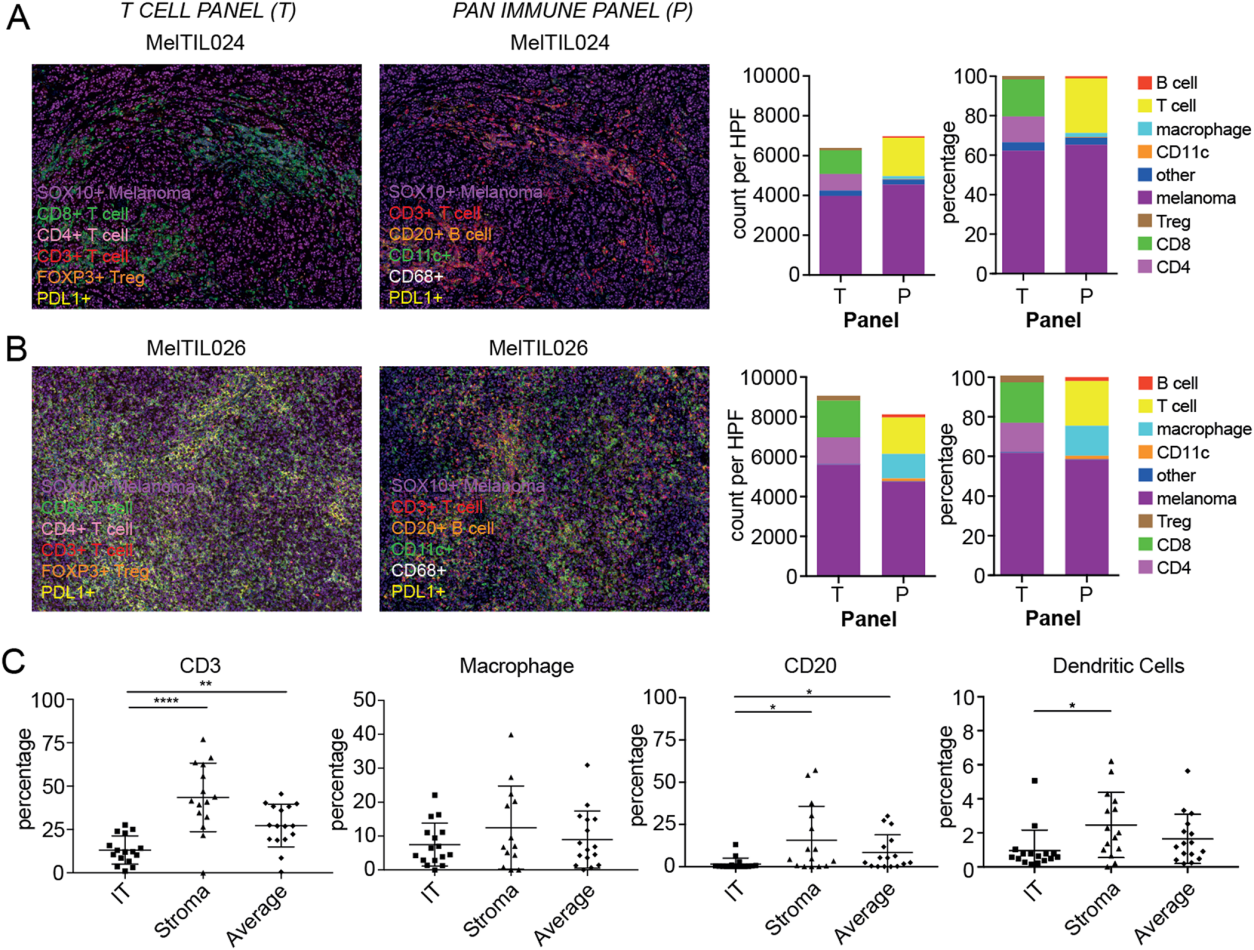
Multiplex IHC reveals the broader immune context of metastatic melanoma. FFPE sections of metastatic melanoma from MelTIL024, s.c. arm metastasis (**A**) and MelTIL026, pelvic lymph node metastasis (**B**) were stained by OPAL for the “T-cell Panel” including SOX10 (melanoma marker), PDL1, CD3, CD8, CD4 and FOXP3 and in the “Pan-Immune Panel” SOX10, PDL1, CD3, CD20, CD68 and CD11c. Stained sections were imaged on the Vectra Automated Imaging System and composite HPF images displayed. The T cell Panel showed the distribution of tumour cells (purple), PDL1 (membranous yellow) on either melanoma or infiltrating APCs, CD8+ T cells (green), CD4+ T cells (pink) and T regulatory T cells (orange nucleus). The pan-immune panel showed distribution of tumour cells (purple), PDL1 (membranous yellow) on either melanoma or infiltrating APCs, CD3+ T cells (red), CD20+ B cells (orange), CD68+ macrophages (white cytoplasmic), and CD11c+ likely dendritic cells (green membranous). High-powered fields were selected based on highest TIL density, and cell populations measured using inform v2.2 software. Immune context quantitation from the T cell panel and pan immune panel was recorded and plotted in Graph Pad PRISM as counts per HPF and % of total cells for each resected metastasis. Collated data for all Mel-TIL samples in the study cohort is depicted in (**C**) including the % of T cells (CD3), B cells (CD20), macrophages (CD68) and CD11c^+^ (likely dendritic cells) in three tumour regions (IT, TM and S) as well as the average of these regions. Statistical analysis (Mann Whitney) was performed across the cohort, significantly different data is represented by *(p < 0.05), **(p < 0.01) and ****(p < 0.0001).

To explore the immune context more broadly we developed a pan-immune OPAL panel comprising CD3 (T cells), CD20 (B cells), CD68 (macrophages), CD11c (likely dendritic cells), SOX10 (melanoma) and DAPI (cell nucleus). We performed both the T cell panel (described above) and the pan-immune panel on serial sections of all melanoma biopsies in the cohort. In Fig. 2, representative high power images of an IT region is shown for both the T cell and pan-immune mIHC panels on melanoma biopsies from patients MelTIL024 (Fig. 2A) and MelTIL026 (Fig. 2B), and MelTIL023 (Supplementary Fig. 5A), and MelTIL006 (Supplementary Fig. 5B). Not surprisingly, melanoma cells were the predominant cells present in MelTIL024 and MelTIL026. However, T cells were also present in large numbers, along with lower numbers of macrophages, B cells and CD11c^+^ cells. Collated data for the whole patient cohort (Fig. 2C) showed significantly increased stromal vs IT T cells, B cells, and CD11c^+^ (likely dendritic) cells. In contrast, no difference in macrophage distribution between IT and stromal regions was observed across the cohort.

### Melanoma multiplex IHC and FACS TIL data are complementary, but numerically different

**Figure 3:**
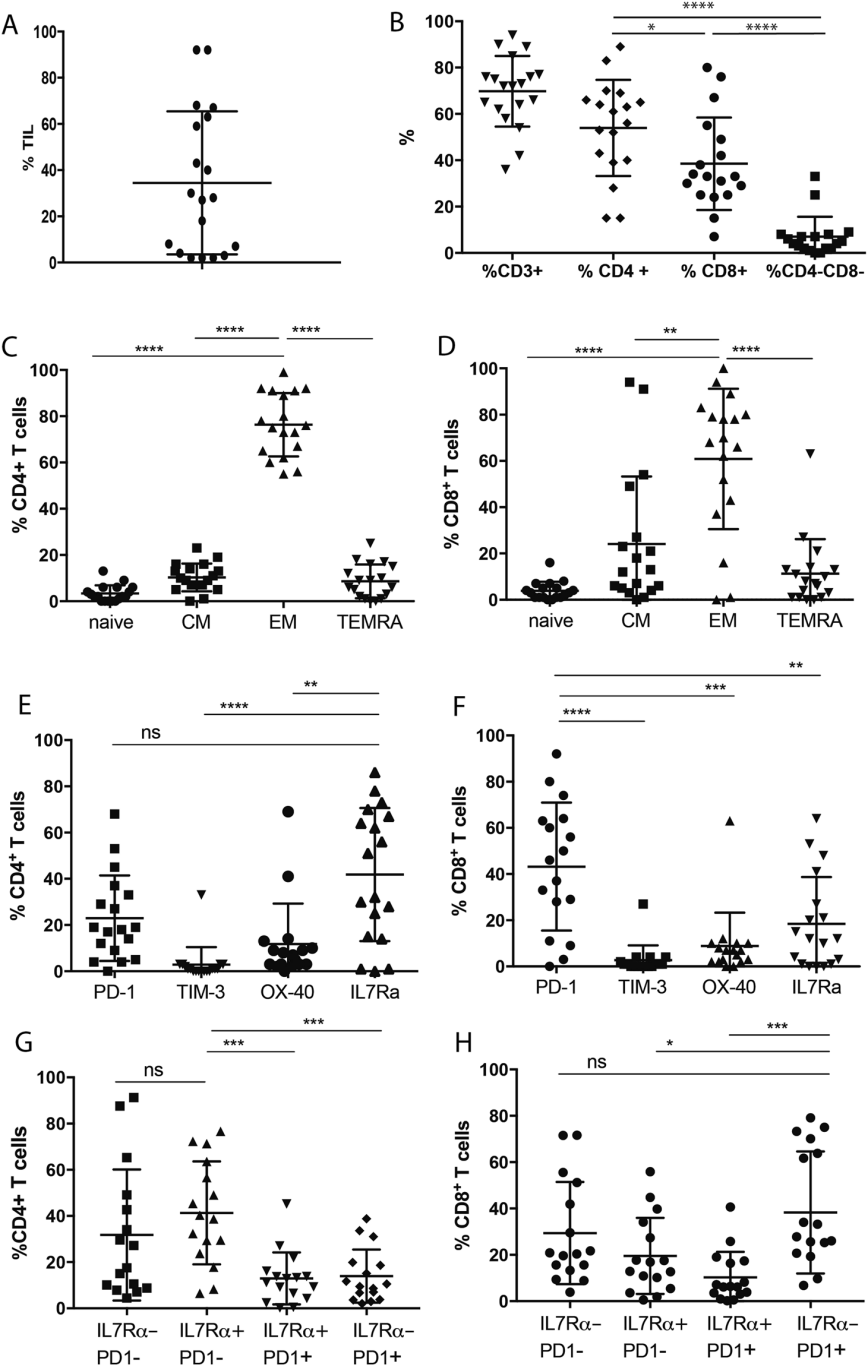
Melanoma TILs. Melanoma metastases were analysed by FACS (gating strategy as per Supplementary Fig. 6) to characterize the T cells present. Viable cells were assessed for (**A**) % TILs (HLA-ABC^+^CD45^+^), and (**B**) T cell subsets. In addition, T cell compartments in (**C**) CD4^+^ T cells, and (**D**) CD8^+^ T cells; immune checkpoints and activation markers in (**E**) CD4^+^ T cells, and (**F**) CD8^+^ T cells, and PD-1 expression by T cell compartments in (**G**) CD4^+^ T cells, and (**H**) CD8^+^ T cells. Finally, relative expression of IL-7Rα and PD-1 was assessed in (**I**) CD4^+^ T cells, and (**J**) CD8^+^ T cells. Data is presented as %+ for each marker; statistical analysis (Mann Whitney) was performed across the cohort, significantly different data is represented by *(p < 0.05), **(p < 0.01), ***(p < 0.001) and ****(p < 0.0001).

In this study, we used the multi-parameter nature of FACS to reveal tumor-associated T cell subset lineage, differentiation and functional polarization (Supplementary Fig. 6). We also examined details of T cell subset immune checkpoint expression to provide information relevant to CBI therapy (Supplementary Fig. 6). This analysis showed the %TILs varied widely between patient tumors (Fig. 3A). T cells were the dominant TIL subset (Fig. 3B), and comprised significantly higher CD4^+^ than CD8^+^ T cells or CD3^+^CD4^−^CD8^−^ T cells. Both CD4^+^ and CD8^+^ T cell subsets were predominantly T effector memory (TEM) (Fig. 3C,D). CD4^+^ T cells heterogeneously expressed PD-1, OX-40 and IL-7Rα, but not TIM-3 (Fig. 3E). In contrast, CD8^+^ T cells preferentially expressed PD-1 than IL-7Rα (new Fig. 3F). All TIL T cell compartments expressed PD-1 (Supplementary Fig. 7), only CD8^+^ TEM expressed significantly higher PD-1 levels than CD8^+^ CD45RA+ T effector memory (TEMRA) cells (Supplementary Fig. 7). Further analysis showed CD4^+^ T cells preferentially expressed IL-7Rα; in contrast CD8^+^ T cells preferentially express PD-1 (Fig. 3G–H). Taken together, the presence of CD8^+^PD-1^+^IL-7Rα^−^, CD4^+^PD-1^+^, and CD4^+^OX-40^+^ T cells indicates activated T cells in the tumor. Also present were CD4^+^IL-7Rα^+^PD-1^−^ and lower numbers of CD8^+^ IL-7Rα^+^PD-1^−^, likely TEM cells trafficking to the tumor site but not responding to antigen. To explore whether TIL parameters common to mIHC OPAL imaging and FACS were comparable, we analyzed paired data from 21 melanoma biopsies. Following the initial TIL assessment (Supplementary Table 2), two samples (MelTIL005 and MelTIL009) were excluded from further analysis due to technical reasons.

**Figure 4:**
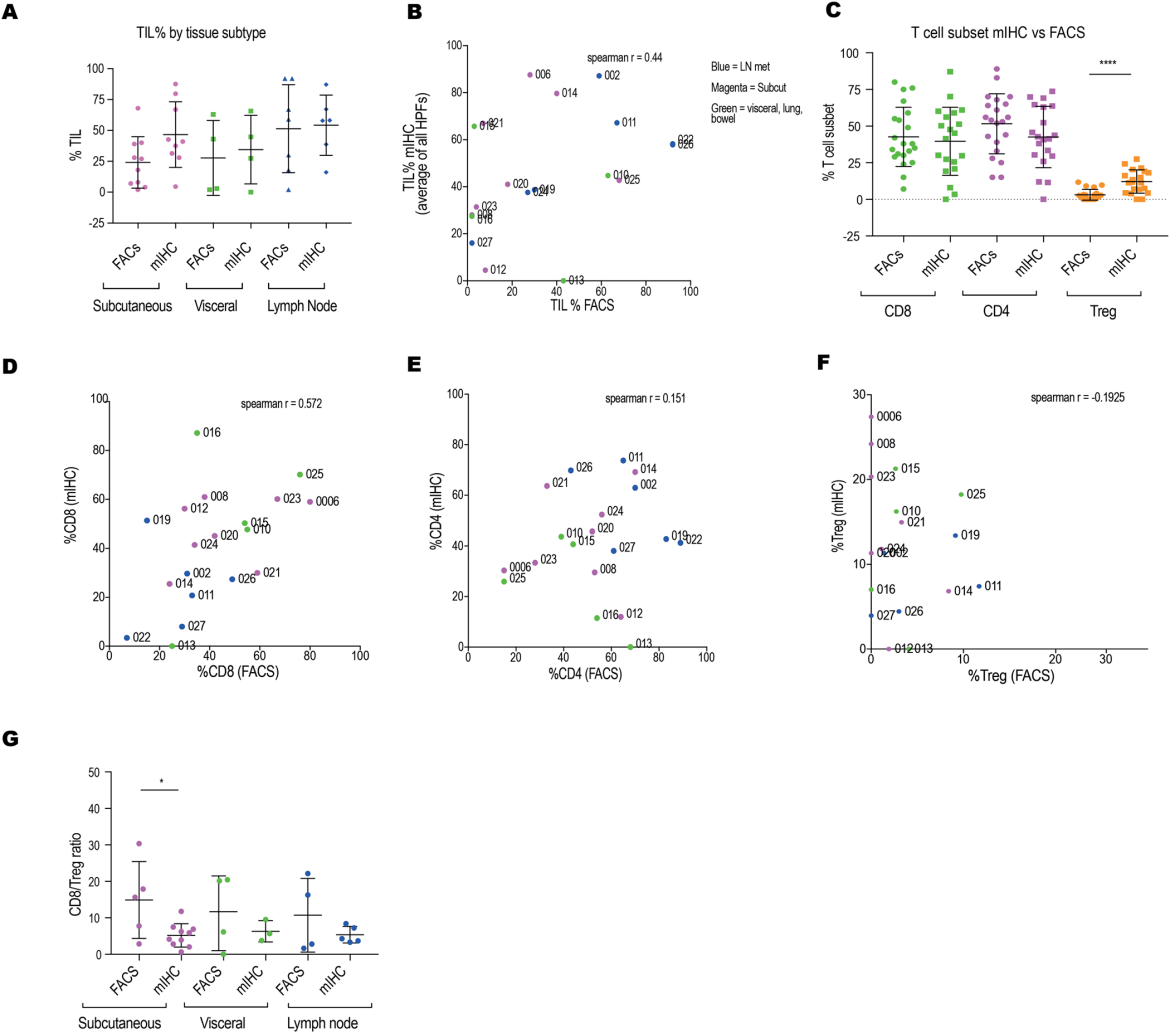
Melanoma stromal distribution of CD4^+^ T cells is a key factor for discrepant TIL data between mIHC and FACS. Melanoma metastases were analysed by FACS and mIHC (as per methods) and the T cell data compared. %TILs were measured by FACS as %(CD45+ HLA− ABC+) of all viable cells (as depicted in Supplementary Fig. 6) and %TILs by mIHC as the %CD3^+^DAPI^+^ cells of total DAPI per HPF, and expressed as an average of multiple HPFs. Shown in (**A**) collated data for the cohort for %TILs measured via FACS vs mIHC, and divided into groups based on metastatic site; (**B**) a direct comparison of paired data for %TILs measured via FACS vs mIHC. Also, the % T cell subsets (CD8^+^, CD4^+^, Treg) measured by FACS vs mIHC in (**C**) for the whole cohort. A comparison of paired data (measured via FACS vs mIHC) in (**D**) %CD8^+^ T cells, (**E**) %CD4^+^ T cells, and (**F**) Treg. Finally CD8:Treg ratio was compared for mIHC vs FACS and grouped according to tissue origin (**G**). Statistical analysis (Mann Whitney) was performed across the cohort comparing mIHC and FACS data, significantly different data is represented by *(p < 0.05) and ****(p < 0.001). Also a Spearman correlative analysis was performed for (**B**) and (**D**–**F**), the r value is provided in each graph.

First, we showed there was no statistical difference in %TILs assessed by FACS vs mIHC (average per HPF) when all %TIL data was grouped into tissue origin (Fig. 4A). However, when we analyzed paired data, a poor correlation was observed between mIHC TIL% (average per HPF) with FACS TIL% (R = 0.44) (Fig. 4B). Within this data set, MelTIL013 and MelTIL022 were extreme outliers that showed no IT TILs by mIHC, but plentiful TILs by FACS (Supplementary Fig. 8). The mIHC analysis of MELTIL013 demonstrated the T cell population to be confined to the stroma and the IT and TM regions were completely bereft of immune cells, whereas the FACS sample from the same biopsy had 33% TILs (Supplementary Fig. 8A–C). MelTIL022 contained plentiful TILs by FACS (92%), but had exclusively distant stromal distribution of TILs by mIHC (Supplementary Fig. 8D–F). Other outliers included MelTIL015 and MelTIL021, both with a higher %TIL by mIHC vs FACS. We next compared the % of T cell subsets (CD8^+^, CD4^+^ and Treg) between mIHC (average % per HPF) and FACS for the whole cohort (Fig. 4C). Using pooled data for the cohort, there was no statistical difference for %CD8^+^ or %CD4^+^ T cells between mIHC or FACS data; however there was a significantly higher %Treg by mIHC compared to FACS (p < 0.005). This significant difference in %Treg occurred in subcutaneous metastases (mIHC vs FACS) (p < 0.01), but not in visceral and lymph node metastases (Supplementary Fig. 9). A paired analysis (mIHC vs FACS) of T cell subsets for each sample (Fig. 4D–F) showed a poor correlation, this varied between T cell subsets analyzed. For CD8^+^ T cells the correlation was (r = 0.572), CD4^+^ T cells (r = 0.151), and Treg (r = −0.192). Finally there was a significant difference in CD8:Treg ratio (assessed by mIHC vs FACS), particularly in subcutaneous melanoma metastases (Fig. 4G).

### Multiplex IHC identified PDL1 expression on melanoma or macrophages was associated with intra-tumoral CD8^+^ T cells

**Figure 5:**
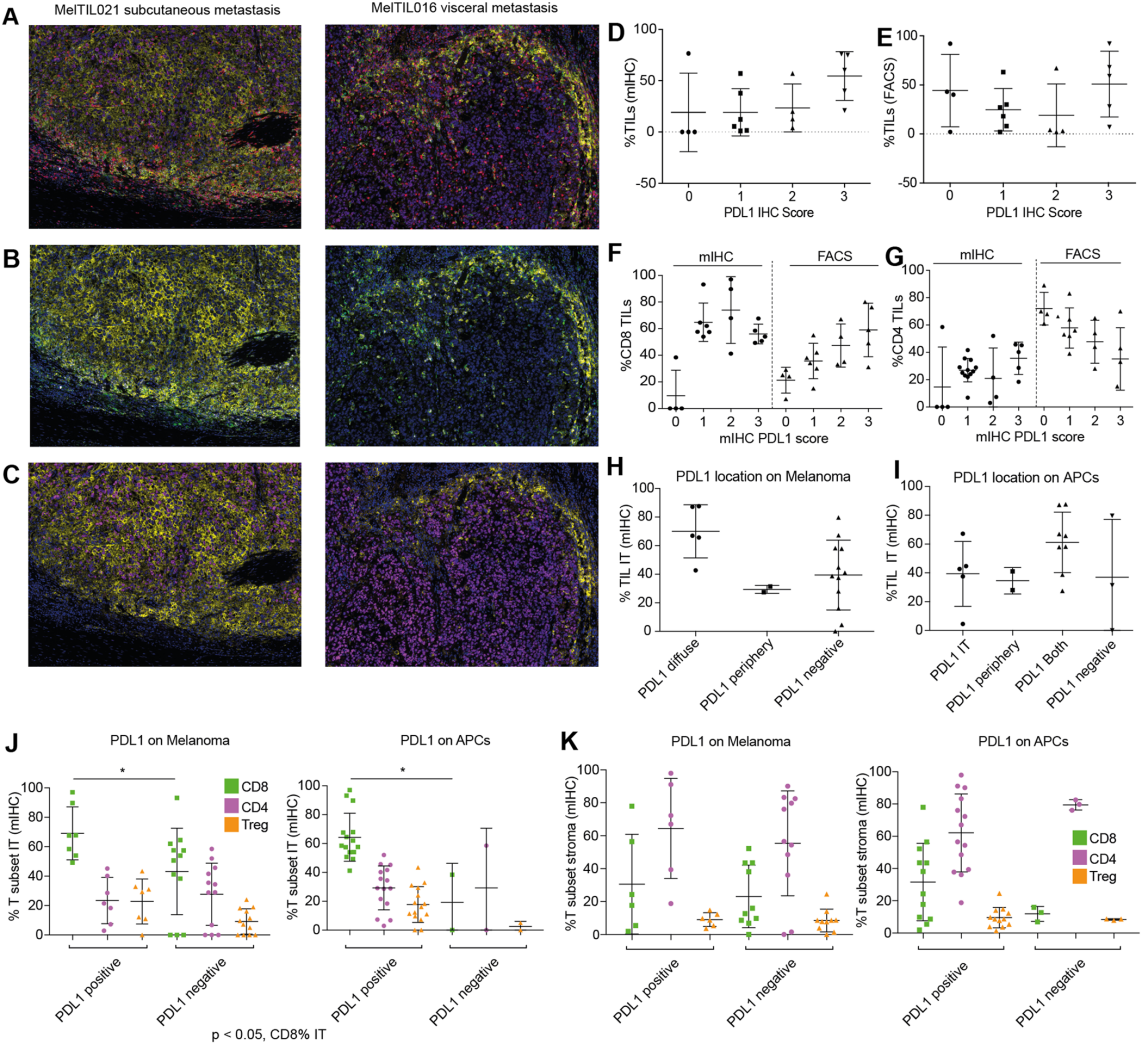
Multiplex IHC describes which cells express PDL1 and their precise location within metastatic melanoma. OPAL stained FFPE sections were imaged on the Vectra Automated Imaging System and cell phenotyping assigned for PD-L1 expression. Colour separation was performed on Inform Software v2.2 to create a composite image (**A**) where CD3 (red), CD20 (orange), CD68 (white), CD11c (green), SOX10 (purple), PDL1 (yellow), DAPI (blue). The composite image was edited to display DAPI and PDL1 with (**B**) CD68 and CD11c or (**C**) SOX10. This combination of images enabled appropriate phenotyping of the melanoma and/or the antigen-presenting cells for PDL1 expression. Using this strategy, all melanoma samples were examined for PDL1 expression. The PDL1 score by mIHC staining (0 = <1%; 1 = >1%, <5%; 2 = >5%, <10%; 3 = >10%) was compared to TIL percentage by mIHC (**D**) and FACS (**E**). The PDL1 score was then compared to the intratumoral (IT) CD8 (**F**) and CD4 (**G**) percentages by mIHC and FACS. Collated data depicted the location of PD-L1 staining for melanoma (diffuse, peripheral or negative) (**H**) and/or macrophages (intra-tumoral, peripheral, both or negative) (**I**) this was plotted against % IT TIL by mIHC. Also correlation between PDL1^+^ cell location plotted against % IT CD8, CD4 and Tregs TILs by mIHC (**J**) as well as the comparison to % stromal CD8, CD4 and Tregs (**K**). Statistical analysis (Mann Whitney) was performed across the cohort comparing mIHC and FACS data, significantly different data is represented by *(p < 0.05).

Both mIHC OPAL panels showed PDL1^+^ cells associated with the immune infiltrate in the IT, TM and stroma regions. In order to resolve clearly whether antigen presenting cells (APC, macrophages and CD11c^+^ likely dendritic cells) or melanoma cells expressed PDL1, a specific analysis protocol was developed (Fig. 5A–C). To do this, the seven color composite images were re-configured to display either APC (CD68, CD11c) and PDL1 fluorescence together (Fig. 5B) or SOX-10^+^ melanoma and PDL1 together (Fig. 5C). Using this approach, MelTIL021 was classified as PDL1-diffuse melanoma with marginal and infiltrating PDL1^+^ macrophages. In contrast, MelTIL016 was classified as PDL1-negative melanoma with marginal and infiltrating PDL1^+^ macrophages. This analysis was applied to the entire cohort and the collated data analysed for the PDL1^+^ cell types and their histological location (Fig. 5D–K). Thus, PDL1^+^ melanoma cells were grouped according to diffuse or tumor periphery location, and PDL1^+^ macrophages into IT or peripheral location. In addition, each melanoma tumor was assigned a PDL1 score based on %PDL1^+^ cells by mIHC (0 < 1%; 1 = 1–5%; 2 = 6–10%; 3 > 10%). Using this dual PDL1 classification system, we first examined whether the frequency of PDL1^+^ cells was associated with the %TIL assessed by mIHC (Fig. 5D) or FACS (Fig. 5E). In all PDL1^+^ cases (15/19), TILs were present, and there was a trend to increased TILs as the PDL1 score increased. However, in a subset of PDL1 negative cases, TILs were also present (1/4 by mIHC and 3/4 cases by FACS). This may indicate a melanoma not responding to IFN-γ^14^, or chronically stimulated T cells unresponsive to antigen^15^.

We next correlated the PDL1 score with the percentage of CD8^+^ or CD4^+^ T cells (Fig. 5F,G). A PDL1 score of 1–3 (14/19 cases) was associated with >40% CD8^+^ IT T cells by mIHC, and PDL1 score was associated with increasing CD8^+^ T cells by FACS (Fig. 5F). In contrast, IT CD4^+^ T cells by mIHC did not correlate with PDL1 score. Interestingly, decreasing CD4^+^ T cells by FACS was associated with increased PDL1 score (Fig. 5G). Because PDL1 expression is known to be induced by IFN-γ secreted by effector T cells, we examined correlations between PDL1^+^ melanoma (Fig. 5H) and macrophages (Fig. 5I) with % IT TIL assessed by mIHC. PDL1^+^ melanoma cells were observed in only 7/19 cases, and of these, 2/7 cases showed PDL1^+^ melanoma cells in the tumour periphery and 5/7 diffuse PDL1^+^ melanoma (Fig. 5H). PDL1^+^ macrophages (FACS or mIHC) were observed in 15/19 cases, they were present in the IT region (5/15 cases), the periphery (2/15 cases) or both IT and periphery (Fig. 5I). We next correlated the PDL1^+^ melanoma or macrophage cell location with %CD8^+^ or %CD4^+^ T cells (mIHC) and their location (IT or stroma). This showed that, irrespective of whether PDL1^+^ expression was on melanoma cells or macrophages, this was associated with significantly higher levels of IT CD8^+^ cells than in samples with negative PDL1- expression (Fig. 5J). In contrast, PDL1 expression was not correlated with stromal CD8^+^ or CD4^+^ T cells or Treg infiltration (Fig. 5K).

#### A model of the immune context of metastatic melanoma

**Figure 6:**
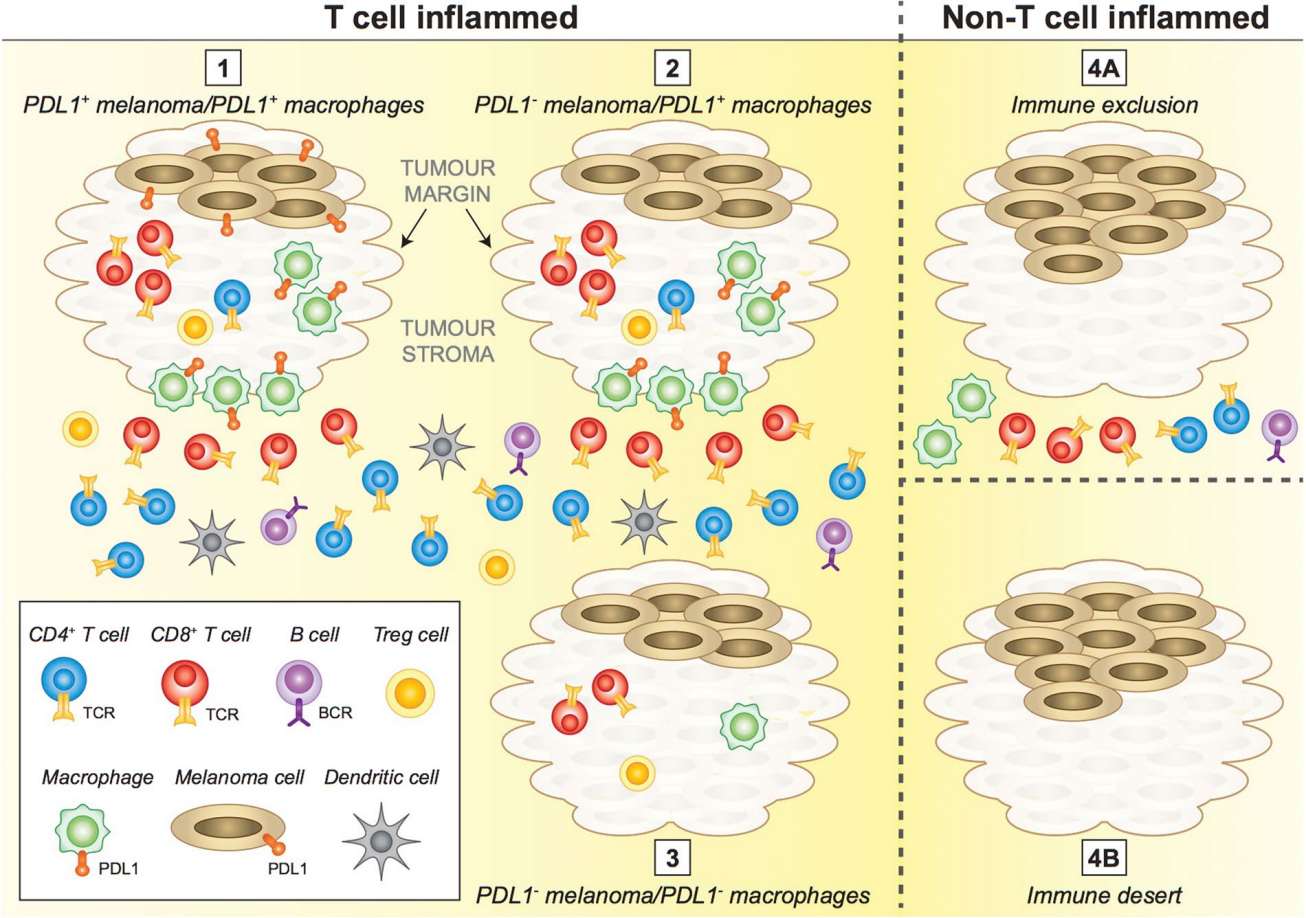
Conceptual diagram to depict metastatic melanoma immune context. Immune context of metastatic melanoma can be categorised into four distinct categories. Categories 1–3 are ‘T cell inflamed’ and characterised by IT CD8^+^ T cells and Treg, plus CD8^+^ T cells at the TM; CD4^+^ T cells, B cells and CD11c^+^ (likely dendritic cells) are in the tumor stroma. Category 1 also has PDL1^+^ melanoma and PDL1^+^ macrophages (IT and at TM). In Category 2, melanoma cells are PDL1^−^, but macrophages are PDL1^+^ (IT and/or TM). In category 3, melanoma and macrophages cells are PDL1^−^. Category 4 includes the T cell excluded (4A) where all immune cells are in the stroma and both melanoma and macrophages are PDL1^−^, or immune desert (4B) where no immune cells are present.

Based on the mIHC and FACS data generated in this study, we propose a model of melanoma immune context that identifies different categories of tumor immune control (Fig. 6). This model incorporates PDL1 expression on melanoma and/or macrophages and their location, with the presence of IT CD8^+^ T cells. We observed ‘hot’ tumors, where IT CD8^+^ T cells were present, in 16/19 patient tumors: 15/19 of these tumors had a PDL1 score of 1–3, and PDL1 was more commonly expressed on macrophages (IT or peripheral) than melanoma cells. The 3/19 patients whose melanoma lacked IT CD8^+^ T cells also were PDL1 negative. The tumor margin was enriched for CD8^+^ T cells; whereas the tumor stroma comprised increased CD4^+^ T cells, B cells and CD11c^+^ cells. Tregs were present IT, TM and the tumor stroma. Thus, our model of metastatic melanoma immune context can be divided into 4 categories. Categories 1–3 contain IT CD8^+^ T cells and can be sub-divided into (1) PDL1^+^ macrophages and PDL1^+^ melanoma; (2) PDL1^+^ macrophages (IT and/or periphery) and PDL1^−^ melanoma, or (3) PDL1^−^ macrophages and PDL1^−^ melanoma. Finally category 4 (eg. MELTIL013 and MELTIL022) displayed complete immune exclusion from the tumor, and either distant immune cells (not part of the tumor stroma) or a cold tumor devoid of immune cells. Categories 1–3 contain CD4^+^ T cells largely in the tumor stroma with increased frequency distant from the TM. Using our immune context model, we assigned individual patient melanoma metastases an immune context number (Supplementary Table 3). For our patient cohort, the spread of melanoma immune context was category 1 (n = 6), category 2 (n = 7), category 3 (n = 3), category 4 (n = 3).

#### Correlation of melanoma immune context and response to checkpoint inhibitors

Prior to surgery, four of the patients in the study cohort had received checkpoint inhibitor (anti-CTLA4, ipilimumab) with one partial response (PR, MelTIL016), two patients with stable disease (SD, MelTIL025 and MelTIL026) and one patient with progressive disease (PD, MelTIL021). All patients subsequently had progressive tumour deposits which were excised for inclusion in this study and examined by FACS and mIHC. There was diversity in the immune categories of these tumours representing immune context categories 1–3.

At various time points following enrolment in this study, 9/21 patients received checkpoint inhibitor therapy including anti-CTLA4 (ipilimumab, n = 3) alone, anti-PD-1 alone (pembolizumab or nivolumab, n = 5) or combination therapy (ipilimumab and nivolumab, n = 1). Responses to anti-CTLA-4 included CR (MelTIL006 and MelTIL008), and a PR (MelTIL014); response to anti-PD-1 alone included CR (MelTIL015 and MelTIL027), PR (MelTIL012 and MelTIL021), and PD (MelTIL010). The one patient (MelTIL013) who received anti-CTLA4 and anti-PD-1 had PD. Of the nine patients who responded to CBI with a CR or PR, six had some evidence of an intratumoral immune response (immune context category 1–3), however MelTIL027 had a CR despite a category 4 A appearance. By contrast, MelTIL010 (category 2) had PD with PD1 therapy given 12 months following his surgical resection and had rapid disease progression with a poor performance status at the time of systemic therapy. Finally, MelTIL013 was category 4B (immune desert) and had PD.

**Figure 7:**
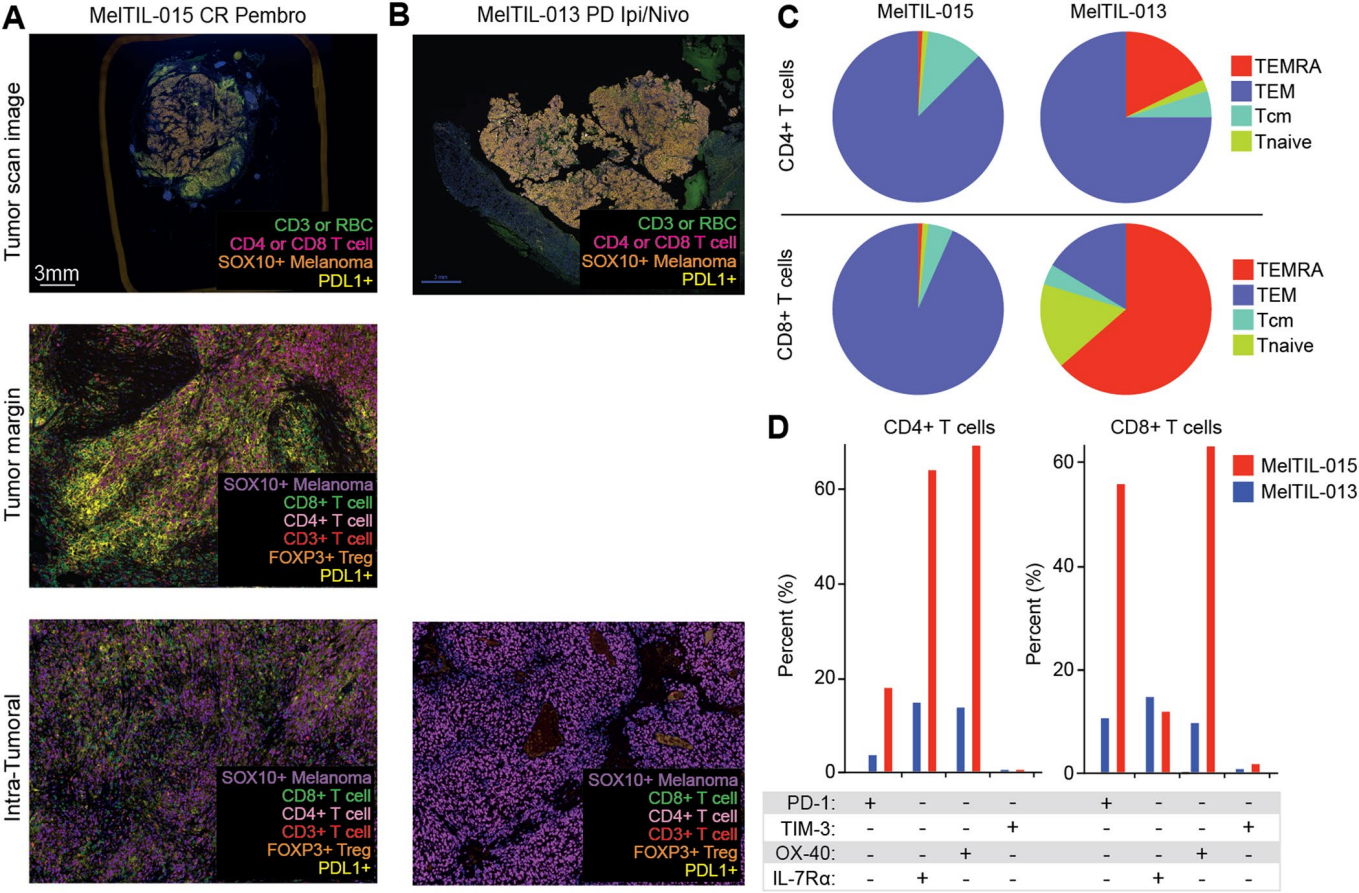
Using metastatic melanoma immune context data to understand checkpoint blockade inhibitor responses. Two metastatic melanoma samples (MelTIL015 and MelTIL013) had divergent responses to CBI. The immune context of each melanoma biopsy was examined by multiplex IHC (**A**,**B**) and FACS (**C**,**D**). Multiplex IHC data is shown for the T cell panel for MelTIL015 (**A**) and MelTIL013 (**B**) including a scanned low power image of the whole biopsy, plus an image at the TM or IT. FACS data was analyzed (as per Supplementary Fig. 6) and T cell differentiation compartments represented in pie chart format for MelTIL015 and MelTIL013 (**C**). In addition, TIL immune checkpoint (PD-1 and TIM-3), activation markers (OX-40) and IL-7Rα expression were represented in column charts for MelTIL015 and MelTIL013 (**D**).

We examined two signature cases to illustrate how FACS and mIHC data could be combined to understand the immune context of individual metastatic melanoma. Both these cases were treated with CBI therapy post surgery; MelTIL015 was treated 9 months following specimen resection and had a CR and MelTIL013 was treated 6 months after surgery and had PD. The mIHC images showed a brisk IT immune infiltrate in MelTIL015, and hot spots at the TM (Fig. 7A)**;** in contrast there were no TILs evident by mIHC in MelTIL013 (Fig. 7B). Despite this apparent lack of TILs in MelTIL013 by mIHC, TIL’s were evident by FACS. The FACS data on T cells from MelTIL015 and MelTIL013 showed divergent differentiation, activation and immune checkpoint expression. MelTIL015 CD4^+^ and CD8^+^ T cells were predominantly TEM, whereas MelTIL013 had a much larger TEMRA compartment (Fig. 7C). MelTIL015 TIL CD4^+^ T cells were 60% IL-7Rα^+^OX-40^+^ and 20% PD-1^+^ cells, whereas CD8^+^ T cells were 60% PD-1^+^OX-40^+^. In contrast TILs from MelTIL013 had low levels of PD-1, OX-40 and IL-7Rα (Fig. 7D). This comprehensive profile indicates that T cells from MelTIL015 were present in large numbers and had penetrated the tumor, that CD4^+^ and CD8^+^ T cells were both activated (PD-1^+^OX-40^+^), and the high level of PDL1 in the tumor suggests secretion of IFN-γ. In addition, MelTIL015 CD4^+^ T cells express high levels of IL-7Rα^+^ suggesting these cells can be supported by the homeostatic cytokine IL-7. In contrast, MelTIL013 CD4^+^ and CD8^+^ TEMRA cells are IL-7Rα^−^ and short-lived. Thus, the immune response to MelTIL015 was vigorous, whist these immune cells were removed along with the tumor, the good clinical response to CBI may reflect systemic immunity in this patient. In contrast, MelTIL013 had terminally differentiated T cells present outside the melanoma TM, these T cells were not activated and there was no PDL1 expression within the tumor suggesting a poor immune response.

## Discussion

Objective assessment of melanoma TILs has traditionally been performed by FACS to derive T cell lineage, activation, differentiation, immune checkpoint expression, and detection of tumor-reactive T cells^16,17^. FACS has been used extensively by immunologists to define immune subsets to an exquisite degree, and this information has been correlated with immune function, both *in vitro* and *in vivo*. Despite this wealth of information, FACS does not reveal where the immune system is in relation to the tumor, an important prognostic indicator in melanoma^8,9^. In contrast, standard histology and H&E staining reveals the histological context of the TILs, but nothing about TIL lineage. Conventional IHC lacks the multi-parameter power to accurately define TIL lineage. The new mIHC technology^13^ addresses this issue and provides multi-parameter cell lineage assessment as well as the histological location of individual tumor and immune subset cells. Thus, mIHC forms a bridge between knowledge derived by multi-parameter FACS (phenotype correlated with immune function) and conventional histology and IHC. In this study we used melanoma biopsies from a cohort of patients with metastatic disease to assess whether mIHC and FACS provided concordant, and where applicable, complementary data. FACS TIL phenotype was an important correlate with mIHC data as it provides objective quantitative data, plus FACS-derived data is the viewpoint from which immunologists understand tumor immune context. Whilst the %TIL was poorly correlated between FACS and mIHC for individual data pairs, this was likely due to differences inherent in the technology (eg cell harvesting for FACS can introduce preferential survival of TILs vs tumor cells), and data analysis (eg live cell gate by FACS vs mIHC DAPI counts). In particular, the %Treg correlation was very poor in subcutaneous metastases and this may reflect poor recovery of these cells from the biopsy for FACS analysis. In our cohort, mIHC data showed unequivocally a preferential accumulation of CD8^+^ T cells within the tumor, and CD4^+^ T cells in the tumor stroma. Whilst FACS cannot provide important information about tissue location of individual immune subsets in relation to the tumor, FACS did provide additional information indicating that TILs comprised largely effector memory T cells, including activated CD8^+^ and CD4^+^ T cells which expressed PD-1 immune checkpoint (and not TIM-3), PD-1^+^ and IL-7Rα^+^ CD8^+^ T cells were mutually exclusive, and OX-40 was only present on CD4^+^ T cells.

In this study, we revealed that mIHC data depicts the human melanoma immune context both visually and numerically. The results showed immune context heterogeneity between tumors as well as within tumors (hotspots), and points to variation in immune escape that was previously under-appreciated. Based on the mIHC data from our patient cohort, we devised an immune context model which categorized metastatic melanoma into four main groups. This model condenses the highly heterogeneous immune context of melanoma into a format which enables comparison between tumors from patients. The model also indicates melanoma-immune context interactions from category one (IT CD8^+^ T cells, PDL1^+^ melanoma and macrophages and high TIL content) through to category four (either immune excluded or immune desert). Subsequent responses to checkpoint blockade in our cohort were related to immune context category numbers 1–3. However, caveats to this interpretation include low patient numbers treated with CBI in our cohort (n = 9), and the biopsy represents a snapshot in time of the dynamic interaction between the immune system and melanoma. Therefore, larger cohorts are required in future studies to validate immune context models which demonstrate predictive value. Recently, patient responses to nivolumab were associated with a reduced neo-antigen load and clonal T cell expansion, suggesting immuno-editing of the tumor by T cells^18^. In this cohort at baseline, the melanoma mutation load was associated with better overall survival and response rate to nivolumab in ipilimumab naïve patients only^18^. In addition, the patient’s overall health and performance status have been shown to strongly influence the likely response to immunotherapy^19^.

To understand how the pre-existing immune contexture of stage III melanoma might influence prognosis, Madore *et al*.^20^ investigated PD-L1 by IHC, and correlated this with tumor non-synonymous mutation (NSM) burden, immune gene expression data and clinical outcome. They showed PD-L1 negative melanoma had a low NSM burden and poor survival. In contrast, PD-L1 positive melanoma was enriched for an immune gene signature characterized by cytotoxic lymphocyte and macrophage genes. Indeed, CD8a^high^ was associated with better melanoma- specific survival and PDL1 positive melanoma. Taken together with our study, immune contexture studies provide pivotal information regarding patient clinical prognosis in advanced stage melanoma.

PDL1 IHC has been used as a screening tool for clinical trial eligibility for patients with anti-PD-1 or anti-PD-L1 therapy. Studies of PD-L1 assays in non-small cell lung cancer (NSCLC) revealed clone 22C3 stains PD-L1 at a higher frequency on tumor vs immune cells^21–24^ whereas clone SP142 stains immune cells at a higher level compared to tumor cells. PD-L1 staining in melanoma is less affected by the PD-L1 Mab used, a study exploring biopsies from primary and metastatic melanoma showed no significant difference in the PDL1 assay used^25^.

It is not clear why the PD-L1 assay performs differently in NSCLC and melanoma. However recent information may provide some insight; a study exploring the binding sites for PD-L1 antibodies revealed SP142 binds to PD-L1 intra- and extra-cellular epitopes on PD-L1 isoform 1. However, PD-L1 isoform 2 lacks AA 19–132, a known binding site for PD-L1 Mab SP142^26^. At this point, it is not known whether PD-L1 isoform 1 or 2 is present in NSCLC vs melanoma tumor cells.

In the melanoma immuno-oncology field, our mIHC study is unique in that it bridges the gap between previously published single color IHC data and FACS. Multi-spectral imaging has been used in primary melanoma to solve the pigment problem in TIL assessment^27^, to predict whether individual melanoma TILs could expand sufficiently for adoptive therapy^28^, to demonstrate that the immunoproteasome is present in TIL-rich regions of melanoma^29^, and to demonstrate the immune context of acquired resistance in metastatic melanoma^30^.

In addition, three important studies used single color IHC to explore melanoma immune context as a predictor of response to CBI^31,32^, or a DC vaccine^33^. These studies correlated location of CD8α^+^ cells^31–33^, immune gene expression profile^31^ and clinical outcome. Our study utilizes a multi-parameter immunophenotype which defines more precisely the type of immune cells present within the tumor, and a model which provides a framework within which the heterogeneity of melanoma immune context can be understood. We expect this type of mIHC data will continue to be refined to understand the immune context of cancer more broadly, and is likely to be an important parameter to assess when deciding patient therapy options. To date, the mIHC technology has proven to be scalable, reliable and lends itself to high throughput. Our experience indicates rapid learning of OPAL staining technology, the imaging instrument, analysis software and bio-informatics. However, key to the success of our project and interpretation of mIHC data was the involvement of an academic pathologist.

## Materials and Methods

Patients undergoing surgical resection of melanoma metastases as clinically indicated were enrolled in a prospective protocol after approval from the Peter MacCallum Cancer Center (PMCC) Human Research Ethics Committee (HREC) under approval no 13/141. All methods performed in this study were carried out in accordance with relevant guidelines and regulations under this approval. All experimental protocols were approved by the PMCC HREC under approval no 13/141. Clinical data was collected prospectively from the patient and missing data was extracted from the medical record. Supplementary Table 1 depicts demographic features of the cohort. Supplementary Table 3 provides details of each patient’s melanoma metastasis including surgical site, biopsy tissue type, melanoma status (wild type, BRAF or NRAS mutation), whether the patient had received checkpoint inhibitor treatment prior to, or after surgery and the clinical response. Immune context details were also included for each melanoma biopsy.

### Preparation of melanoma biopsy cell suspension

All patients enrolled in the study had macroscopic disease. Following tumor excision, a representative fragment of tumor measuring approximately 1 cm^3^ was transferred fresh and sterile to the laboratory for study. All surrounding non-tumor tissue was macroscopically removed from the tumor. Tumor tissue was divided into segments and either placed in neutral buffered formalin for processing to formalin fixed paraffin embedded blocks, or a single cell suspension was created from the melanoma biopsy using a modified published protocol^11^. Briefly, tumor was initially divided into segments and then finely diced into RPMI1640 containing 20% fetal bovine serum, 1 mg/ml collagenase type 4 (Worthington biochemical, Lakewood, NJ), 30 U/ml DNase (Sigma-Aldrich Pty Ltd, Sydney, NSW, Australia), 10 ug/ml gentamicin sulfate (Pfizer Australia, West Ryde, NSW, Australia) and incubated for 30 minutes at 37 °C on a rocker. Digested tumor pieces were teased through a 100 μm sieve, the sieve irrigated with DPBS and the cells collected into a 50 ml conical tube. Pelleted cells were resuspended in RP-10 and used for FACS analysis.

### Antibody labeling cells for FACS

Antibodies used in this study included CD45-BV510, CD127-BV421, CD8-BV605, OX-40-PECy7, PD-1-BV785, CCR7-BV711, CD3-BV711, CD4-BV421 (Biolegend, San Diego, CA); HLA-ABC-PE, CD3-APCH7, CD4-APC, CD45RA-FITC, CD25-APC (BD Biosciences, San Diego,CA), Tim-3 PerCP-EF710, CD127-PECy7 and FoxP3-PE (e-Bioscience, San Diego, CA). Harvested cells were labeled with monoclonal antibodies for 30 minutes at 4 °C in FACS buffer (2% fetal bovine serum in DPBS), washed twice in FACS buffer, then fixed in 2% paraformaldehyde/DPBS. Panel 1 (‘Adaptive’ panel): included CD45 and HLA-ABC to discriminate TIL’s from other cells in the cell suspension, T cell markers CD3, CD4 and CD8, T cell activation markers OX-40 and CD127 (IL-7Rα), activation/exhaustion markers PD-1- and TIM-3 and the T cell differentiation markers CCR7 and CD45RA were also included. Panel 2: T-Regulatory/T cell differentiation panel included CD45, HLA-ABC, CD3, CD4, CD25, CD127 and FoxP3. Cells were stained by direct method (as above for panel 1), then fixed and permeabilised, and stained with the FoxP3 antibody using the e-Bioscience fix and permeabilization reagents according to the manufacturer’s protocol (e-Bioscience, San Diego, CA). In both panels viable cells were revealed using the fixable UV-excitable viability dye (UV-blue, Invitrogen). Multi-parameter FACS data was acquired on the BD LSR II FACS instrument (BD Biosciences, San Diego, CA) and data analyzed using FlowJo v10 software (Treestar Inc. Seattle, CA). A representative example of the gating strategy for panel 1 to define TIL’s and then T cell differentiation, expression of activation and suppressor molecules is shown in Supplementary Fig. 6.

### Multiplex immunohistochemistry (OPAL™) staining protocol, image acquisition and data analysis

The remainder of the tumor sample was formalin fixed and paraffin embedded (FFPE). A pathologist (AC) evaluated H&E stained slides from each FFPE block and selected the one with the largest area of viable tumor cells. Subsequently, 3 µm thick sections of FFPE tissue on super frost plus slides were deparaffinised and rehydrated by serial passage through changes of xylene and graded ethanol for multiplex immunohistochemistry staining with the T cell Panel and Pan Immune Panel. All slides were subjected to a primary heat-induced epitope retrieval (HIER) in 1 mM EDTA buffer, pH 8.0 125 °C for 3 minutes. Subsequent HIERs were dependent on antibody used and performed in the microwave at 90 °C, 10% power for 15 minutes. Antibodies used included rabbit monoclonal CD4 (Spring Bioscience, clone SP35, 1/100, high pH retrieval), rabbit monoclonal CD3 (Spring Bioscience, clone SP7, 1/1000, low pH retrieval), mouse monoclonal CD8 (Thermofisher, clone 4B11, 1/1000, high pH retrieval), rabbit polyclonal FOXP3 (BioSB, 1/100, high pH retrieval), rabbit monoclonal PD-L1 (Spring Bioscience, clone SP142, 1/1000, high pH retrieval), mouse monoclonal SOX10 (Biocare Medical, clone BC34, 1/200, low pH retrieval), mouse monoclonal CD20 (DAKO, clone L26, 1/500, low pH retrieval), mouse monoclonal CD68 (Thermofisher, clone 514H12, 1/200, high pH retrieval), and rabbit monoclonal CD11c (BioSB, clone EP157, 1/2000, low pH retrieval). Endogenous peroxidase in tissues was blocked by incubation of slides in 0.3% hydrogen peroxide solution after incubation with primary antibody. Immunofluorescent signal was visualized using the OPAL™ 7-color fIHC kit (Perkin Elmer, MA) TSA dyes 520, 540, 570, 620, 650, and 690, counterstained with Spectral DAPI. All slides were imaged on the Vectra® 3.0 Automated Quantitative Pathology Imaging System, 200 slide (Perkin Elmer, MA). Color separation, Tissue and Cell Segmentation, and Cell Phenotyping were performed on inForm® Software v2.2 (Perkin Elmer, MA) to extract image data. Slides were examined for the presence of tumor infiltrating lymphocytes within the tumor parenchyma (tumor), and the tissue surrounding the tumor (tumor margin) and distant stroma.

### Multiplex IHC data analysis

All slides were scanned at 10X magnification in order to select for high-powered imaging at 20X (resolution of 0.5 µm per pixel) using Phenochart (Perkin Elmer MA). High powered images included the following: intratumoral zones with dense lymphocytic infiltrate, tumor margin zones, and distant stromal zones to provide an overview of the metastatic tumor microenvironment to compare with flow cytometry analysis of the same samples. An algorithm was designed based on pattern recognition of SOX10-positive areas (tumor) and SOX10-negative areas (stroma, tumor margin). Cell segmentation was done based on all cells counter-stained with DAPI. Cell phenotyping on inForm® was performed by selecting at least 5 representative cells per phenotype, performing reiterations as required until at least 20 representative cells per phenotype were selected. Batch analysis was performed on all high-powered images of melanoma metastases using the same algorithm designed on one representative image per metastasis, to account for staining variability and intensity between patients. A TIL scoring for TIL distribution^34^ was performed on the 10X pre-scanned images of each patient and compared to the TIL score performed by an in-house pathologist on H&E stained sections of the same melanoma metastases (Supplementary Table 2). One high-powered image within the tumor parenchyma with the highest TIL density was used to grade TIL density along with OPAL TIL count and percentage. Multiple images from tumor parenchyma (IT), tumor margin (TM), and distant stroma were quantified and an average taken to compare OPAL TIL count and percentage to FACS results on the same patients (Supplementary Fig. 3). Tumor margin referred to the region immediately adjacent to the SOX10^+^ cell perimeter to 100 μm, and distant stroma regions were SOX10-negative.

### Distance Calculation from Tumor Margin

Distance calculation measurements were performed using MetaMorph® Microscopy Automation and Image Analysis Software (Molecular Devices CA). Tissue segmented images were loaded and an object mask of the tumor segment created. A Euclidean distance mask was then drawn in the stromal region from the tumor segment(s). Depending on the stromal cell type of interest (CD8, CD4, or Tregs), an object mask of each cell was created and applied to the Euclidean distance mask, with intensity counts converted to a measurement of distance from the tumour edge (Supplementary Fig. 10).

### Statistical analysis

Statistical analyses included Mann Whitney test and Spearman correlation analysis. These tests were performed on immune subset data derived via mIHC or FACS to define significant differences in immune subset distribution (Mann Whitney), or differences in mIHC vs FACS data (Spearman correlation). Details of significant differences between data groups have been described in each figure legend.

## Electronic supplementary material

Supplementary File

Supplementary Tables
