# Supplementary material for: Multiplex immunohistochemistry accurately defines the immune context of metastatic melanoma
Source: Sci Rep. 2018 Jul 24;8:11158. doi: 10.1038/s41598-018-28944-3 (PMC6057961; doi:10.1038/s41598-018-28944-3)
Supplement: Supplementary file 1 — Supplementary File [file 41598_2018_28944_MOESM1_ESM.pdf]

Multiplex immunohistochemistry accurately defines the immune context of metastatic melanoma

Halse H[1#], Colebatch AJ[2#], Petrone P[1], Henderson MA[1], Mills J [1,3], Snow H[3], Westwood JA[1], Sandhu S[2,4], Raleigh JM[2], Behren A[5,7], Cebon J[5,7], Darcy PK[1,4], Kershaw MH[1,4], McArthur GA[2], Gyorki DE[1,3,6*] and Neeson PJ[1,4*].

Affiliations:

(1) Cancer Immunology Research (2) Division of Cancer Medicine Melanoma Program (3) Division of Cancer Surgery

Peter MacCallum Cancer Centre, 305 Grattan Street, Melbourne, 3000.

(4) Sir Peter MacCallum Department of Oncology, The University of Melbourne, Parkville, Victoria, 3052, Australia.

(5) Olivia Newton John Cancer Research Institute, Heidelberg, Victoria, 3084, Australia.

(6) Department of Surgery, University of Melbourne, Parkville, Victoria, 3052, Australia.

(7) School of Cancer Medicine, La Trobe University, Bundoora, 3086, Australia

* denotes equal senior author

# denotes equal first author

Corresponding author: paul.neeson@petermac.org

Supplementary Figure 1. Multiplex IHC depicts the heterogeneous immune context of s.c. metastatic melanoma.

Subcutaneous melanoma metastasis biopsies from patients MelTIL006 (A) and MelTIL024 (B) were examined on serial FFPE sections by H&E, and mIHC. For mIHC, FFPE sections were stained by OPAL with the “T cell panel” for SOX10 (purple), PDL1 (yellow), CD3 (red), CD8 (green), CD4 (pink) and FOXP3 (orange). Stained sections were imaged on the Vectra Automated Imaging System and a composite image displayed. Shown are the composite images for the entire biopsy (10X magnification), plus a select region (high T cell region) examined by high power 20X magnification (blue box). The TIL density and distribution is shown for each low power mIHC composite image.

Supplementary Figure 2. Multiplex IHC depicts the heterogeneous immune context of lymph node metastatic melanoma.

Melanoma metastasis biopsies from lymph node of patients MelTIL022 (A) and MelTIL026 (B) were examined on serial FFPE sections by H&E, and mIHC. For mIHC, FFPE sections were stained by OPAL with the “T cell panel” for SOX10 (purple), PDL1 (yellow), CD3 (red), CD8 (green), CD4 (pink) and FOXP3 (orange). Stained sections were imaged on the Vectra Automated Imaging System and a composite image displayed. Shown are the composite images for the entire biopsy (10X magnification), plus a select region examined by high power (blue box). The TIL density and TIL distribution is shown for each low power mIHC composite image.

Supplementary Figure 3. Multiplex IHC depicts the heterogeneous immune context of visceral metastatic melanoma.

Visceral melanoma metastasis biopsies from patients MelTIL010 (A) and MelTIL015 (B) were examined on serial FFPE sections by H&E, and mIHC. For mIHC, FFPE sections were stained by OPAL with the “T cell panel” for SOX10 (purple), PDL1 (yellow), CD3 (red), CD8 (green), CD4 (pink)

and FOXP3 (orange). Stained sections were imaged on the Vectra Automated Imaging System and a composite image displayed. Shown are the composite images for the entire biopsy (10X magnification), plus a select region examined by high power (blue box). The TIL density and TIL distribution is shown for each low power mIHC composite image.

Supplementary Figure 4. Analysis pathway from raw image to phenotyping

Colour separation is performed on Inform Software v2.2 to create a composite image (A). Cell segmentation is performed on Inform Software v2.2 by DAPI counterstain (B), followed by phenotyping using lineage specific markers (CD3+, CD4+, CD8+, FOXP3+, and SOX10+) (C).

For Tumour Margin images, a tissue segmentation step is performed prior to cell segmentation to delineate tumour versus stromal regions (D & E).

Supplementary Figure 5. Multiplex IHC reveals the broader immune context of metastatic melanoma.

FFPE sections of metastatic melanoma from MelTIL023, metastasis (A) and MelTIL006, lymph node metastasis (B) were stained by OPAL for the "T-cell Panel" including SOX10 (melanoma marker), PDL1, CD3, CD8, CD4 and FOXP3 and in the "Pan-Immune Panel" SOX10, PDL1, CD3, CD20, CD68 and CD11c. Stained sections were imaged on the Vectra Automated Imaging System and composite HPF images displayed. The T cell Panel showed the distribution of tumour cells (purple), PDL1 (membranous yellow) on either melanoma or infiltrating APCs, CD8+ T cells (green), CD4+ T cells (pink) and T regulatory T cells (orange nucleus). The Pan-Immune Panel showed distribution of tumour cells (purple), PDL1 (membranous yellow) on either melanoma or infiltrating APCs, CD3+ T cells (red), CD20+ B cells (orange), CD68+ macrophages (white cytoplasmic), and CD11c+ dendritic cells (green membranous). High-powered fields were selected based on highest TIL density, and –cell populations measured using inform v2.2 software. Quantification from the T cell

panel (A) and Pan Immune panel (B) were recorded and plotted in Graph Pad PRISM as counts per HPF and percentage of total cells for each resected metastasis.

Supplementary Figure 6. FACS analysis of melanoma TILs.

FACS analysis of a melanoma metastasis with "brisk" immune infiltrate (MelTIL011), and low immune infiltrate (MelTIL016). A sequential gating strategy was used to define single cells, viable cells and then %TILs as $CD45^{+}HLA\text{-}ABC^{+}$. $CD3^{+}$ T cells were then gated on TILs, $CD4^{+}$ and $CD8^{+}$ T cells were assessed for expression of chronic TCR activation markers PD-1 and TIM-3, the activation markers OX-40 and CD127 (IL-7Rα). In addition, TILs were assessed for T cell differentiation using CD45RA and CCR7 ($CD45RA^{+}CCR7^{+}$ naive, $CD45RA^{+}CCR7^{-}$ TEMRA, $CD45RA^{-}CCR7^{-}$ effector memory and $CD45RA^{-}CCR7^{+}$ central memory).

Supplementary Figure 7. PD-1 is expressed in all T cell differentiation compartments of melanoma TILs.

FACS analysis of each melanoma metastasis was performed as per the methods, using the gating strategy defined in Supplementary Figure 1. T cell differentiation compartments (CM, TEMRA and EM) were analysed for PD-1 expression levels by (A) $CD4^{+}$ T cells and (B) $CD8^{+}$ T cells. Data is presented as %PD-1+ for each T cell differentiation compartment. Statistical analysis (Mann Whitney) was performed across the cohort, and designated either ns (not significant) or significantly different data by ** ($p<0.01$).

Supplementary Figure 8. Outlier assessment

Two examples in which there was a discrepancy in TIL percentage measurements by FACS compared to mIHC are shown. Mel-TIL 013 is an example of sampling error, Mel-TIL 013 mIHC low power image (A) and a HPF example (B) showed zero TILs; in contrast plentiful TILs (CD45+HLAABC+ cells) were present by FACS (C). MelTIL022 is an example of immune exclusion

where TILs were present exclusively in the stroma. MelTIL022 mIHC low power field (D) and a mIHC HPF example (E) showed all TILs in the tumour stroma; in contrast plentiful TILs were present by FACS (F).

Supplementary Figure 9. T cell subset comparison in samples grouped according to tissue origin.

T cell subset analysis (FACS and mIHC) was performed on segments of the same melanoma metastasis. Shown is data for the entire cohort plotted as % T cell subset (CD4$^+$, CD8$^+$ and Treg) and then grouped according to tissue of origin.

Supplementary Figure 10. Distance calculation measurements

Distance calculation measurements were performed using MetaMorph® Microscopy Automation and Image Analysis Software (Molecular Devices CA). Tissue segmented images were loaded and an object mask of the tumour segment created (A). A Euclidean distance mask was then drawn in the stromal region from the tumour segment(s) (B). Depending on the stromal cell type of interest (CD8, CD4, or Tregs), an object mask of each cell was created and applied to the Euclidean distance mask, with intensity counts converted to a measurement of distance from the tumour edge (C).

A

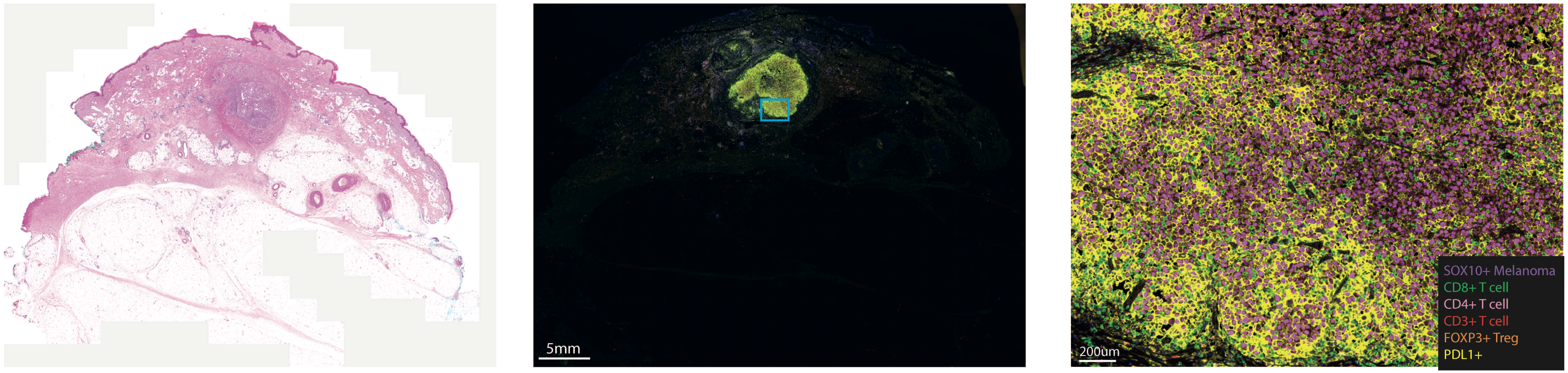

MelTIL006 Subcutaneous Metastasis 10X High Powered Pre-Scan
TIL Distribution = 2; TIL Density = 2

MelTIL006 Subcutaneous Metastasis 20X High Powered Multispectral Image (MFI)

B

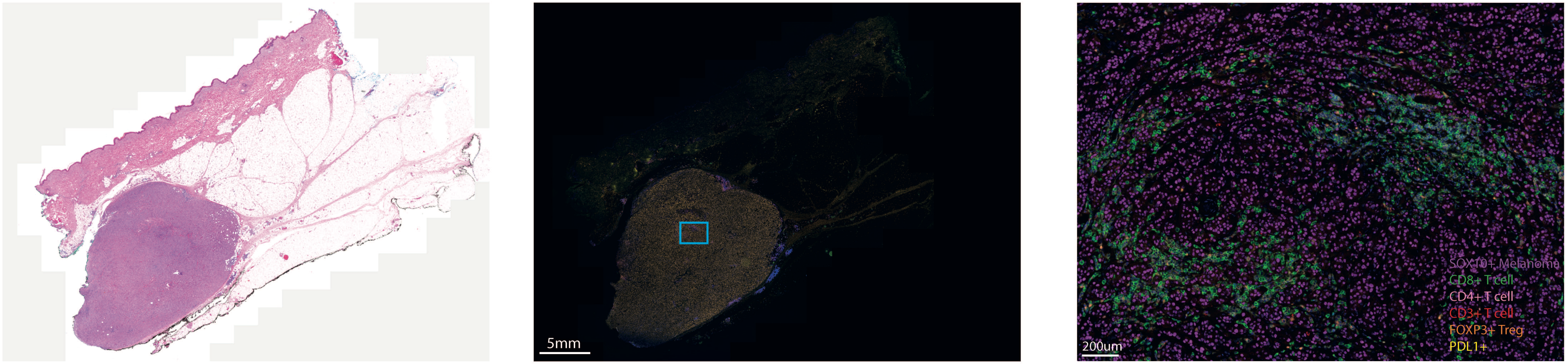

MelTIL024 Subcutaneous Metastasis 10X High Powered Pre-Scan
TIL Distribution = 1; TIL Density = 1

MelTIL024 Subcutaneous Metastasis 20X High Powered Multispectral Image (MFI)

Supplementary Figure 1

A

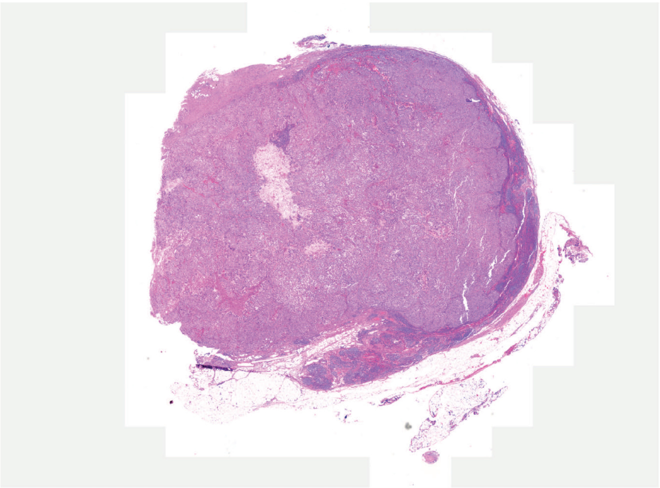

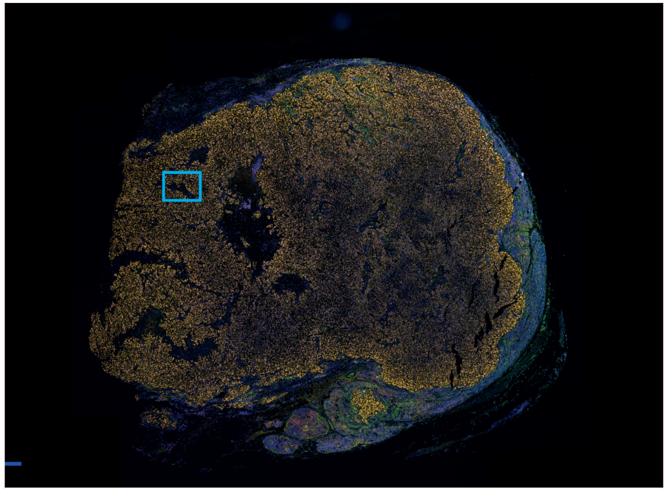

MelTIL022 Lymph Node Metastasis 10X High Powered Pre-Scan
TIL Distribution = 0; TIL Density = 0

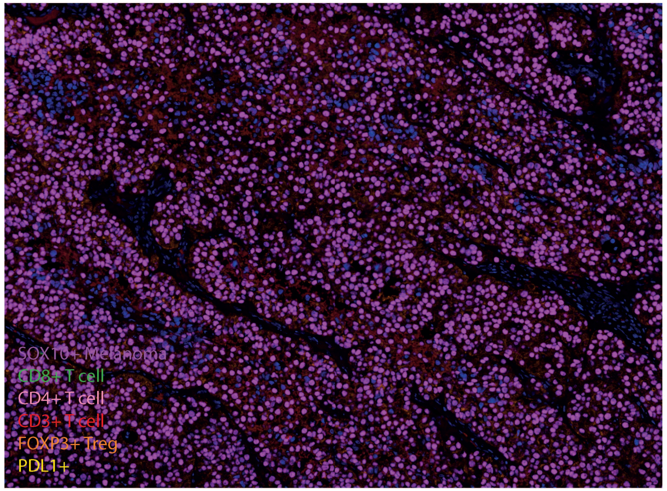

MelTIL022 Lymph Node Metastasis 20X High Powered Multispectral Image (MFI)

B

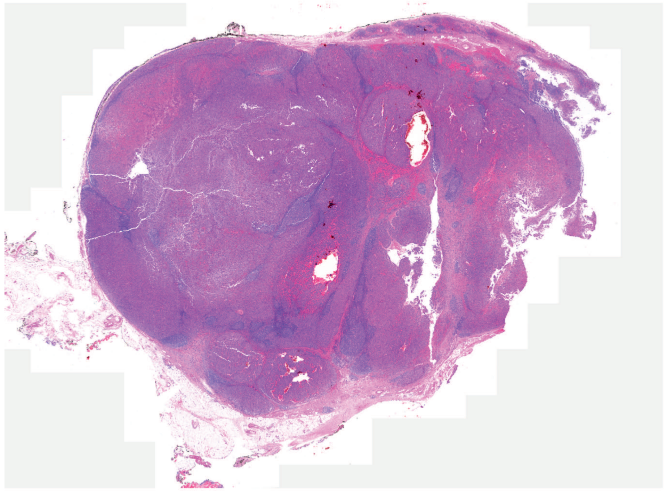

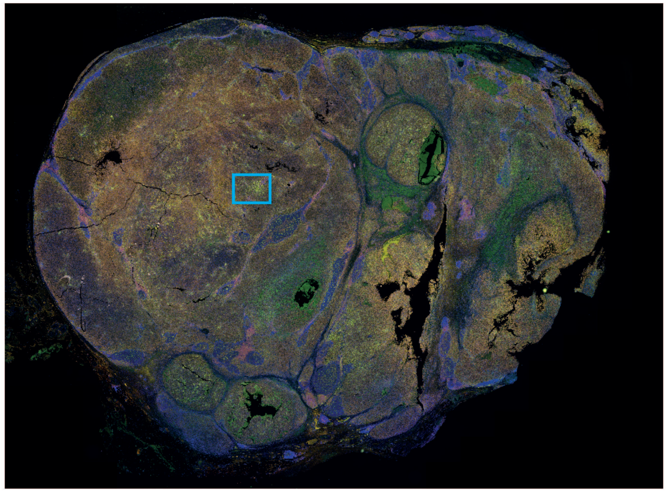

MelTIL026 Lymph Node Metastasis 10X High Powered Pre-Scan
TIL Distribution = 2; TIL Density = 3

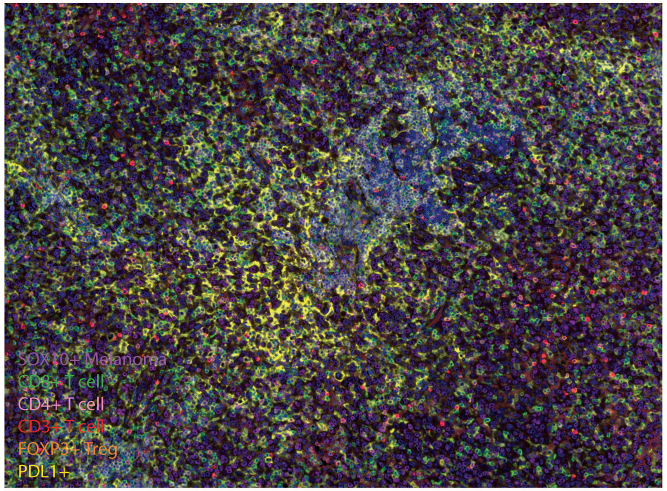

MelTIL026 Lymph Node Metastasis 20X High Powered Multispectral Image (MFI)

Supplementary Figure 2

A

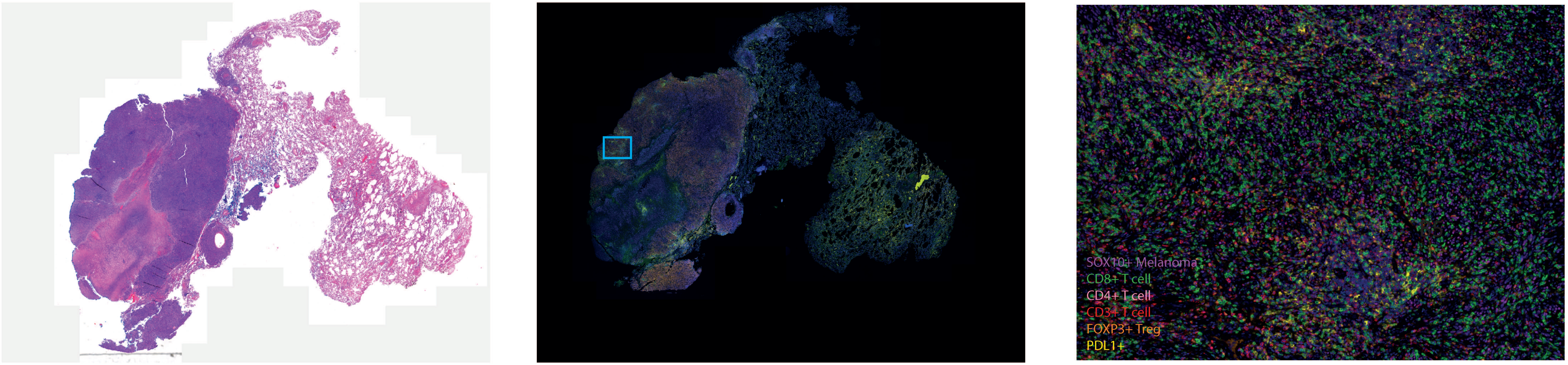

MelTIL010 Visceral Metastasis 10X High Powered Pre-Scan
TIL Distribution = 2; TIL Density = 3

MelTIL010 Visceral Metastasis 20X High Powered Multispectral Image (MFI)

B

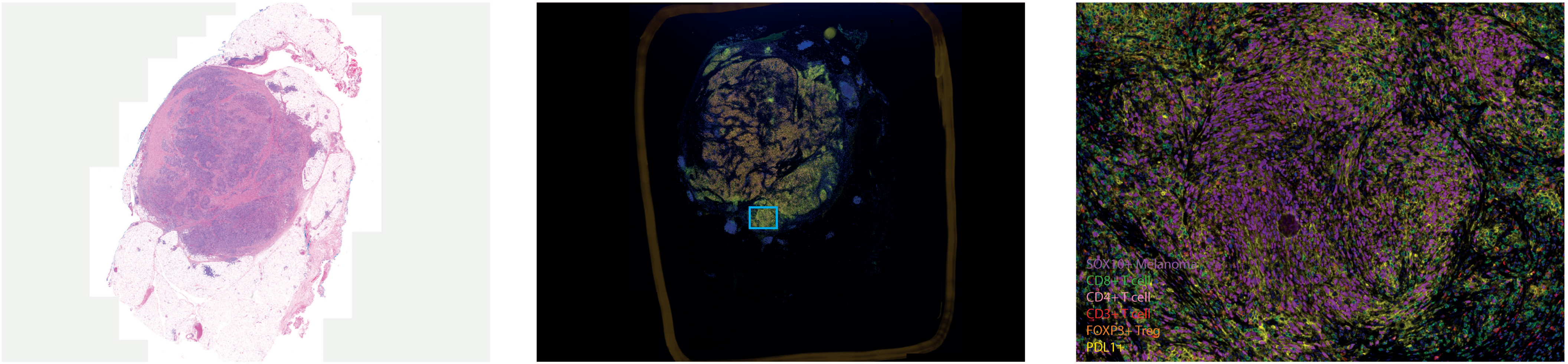

MelTIL015 Visceral Metastasis 10X High Powered Pre-Scan
TIL Distribution = 2; TIL Density = 3

MelTIL015 Visceral Metastasis 20X High Powered Multispectral Image (MFI)

Supplementary Figure 3

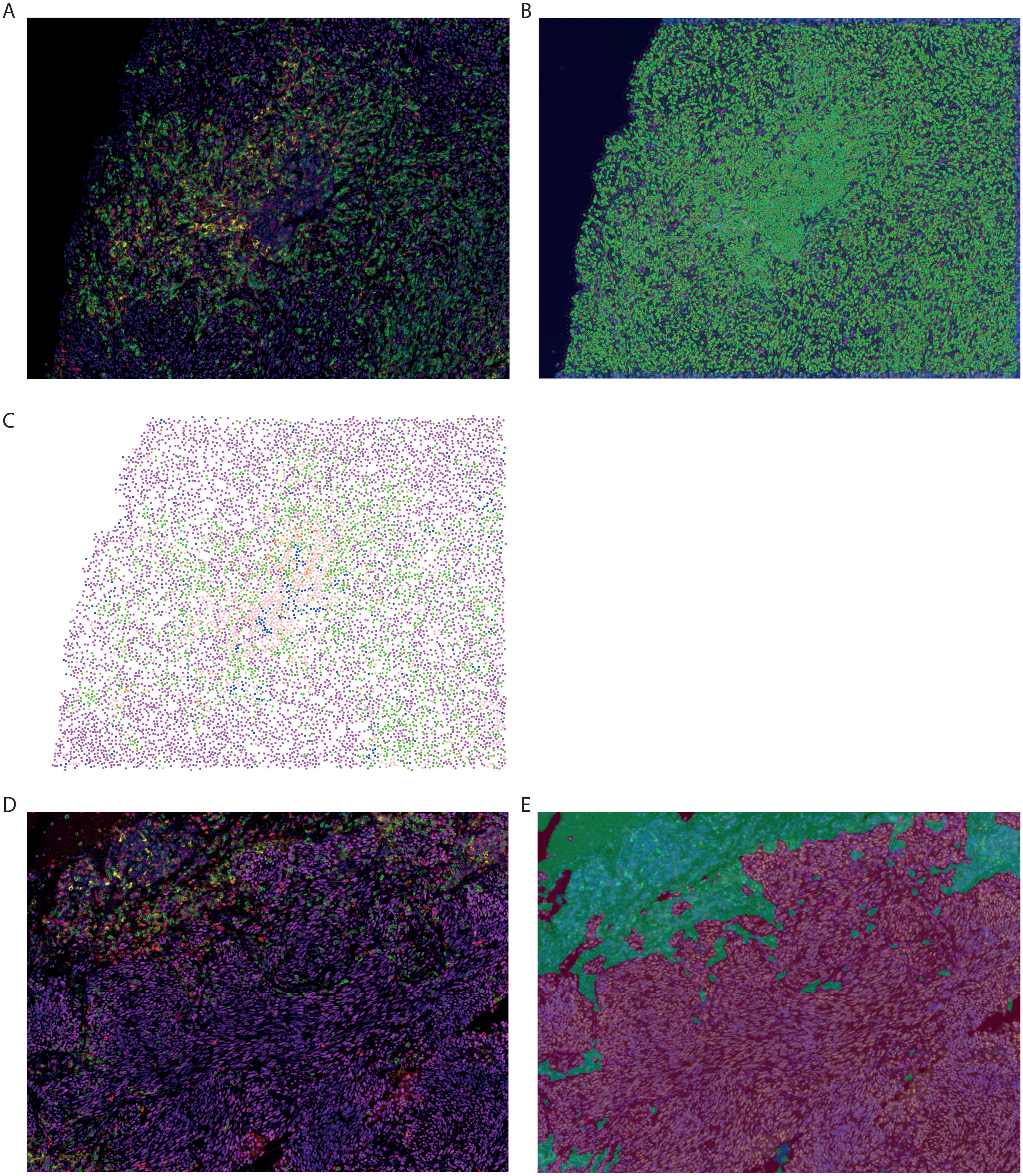

Supplementary Figure 4. Inform tissue segmentation and analysis

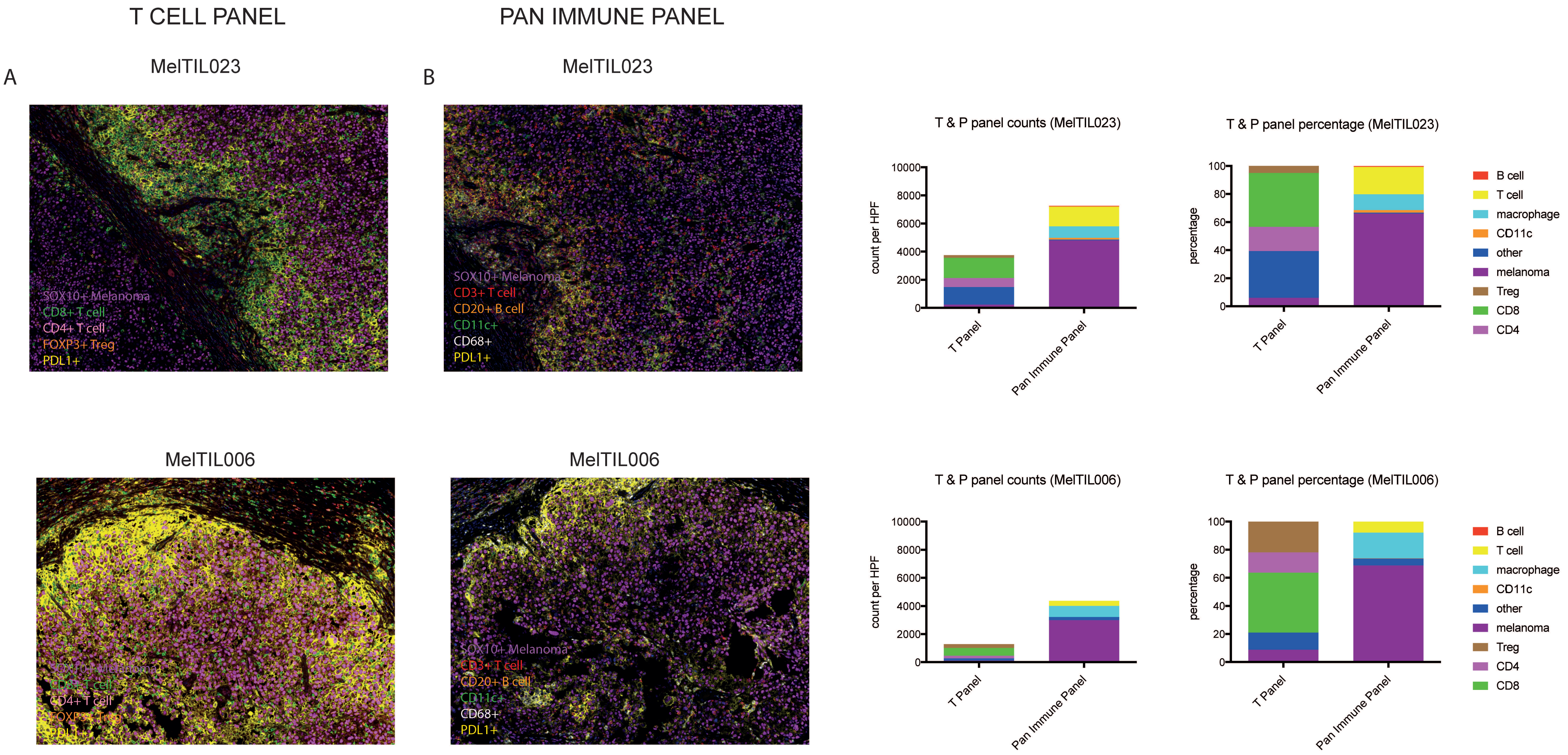

Supplementary Figure 5

MelTIL011

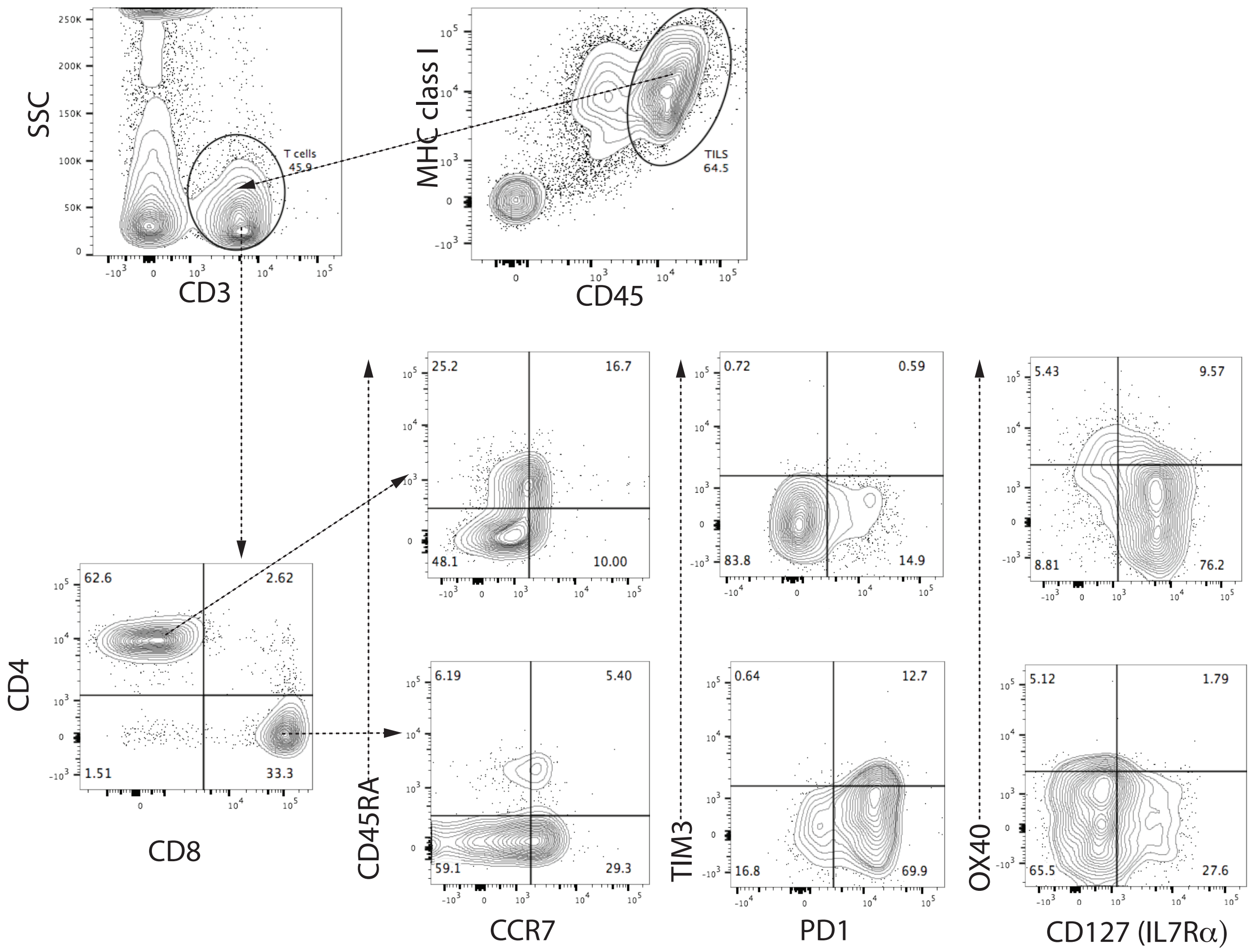

MelTIL016

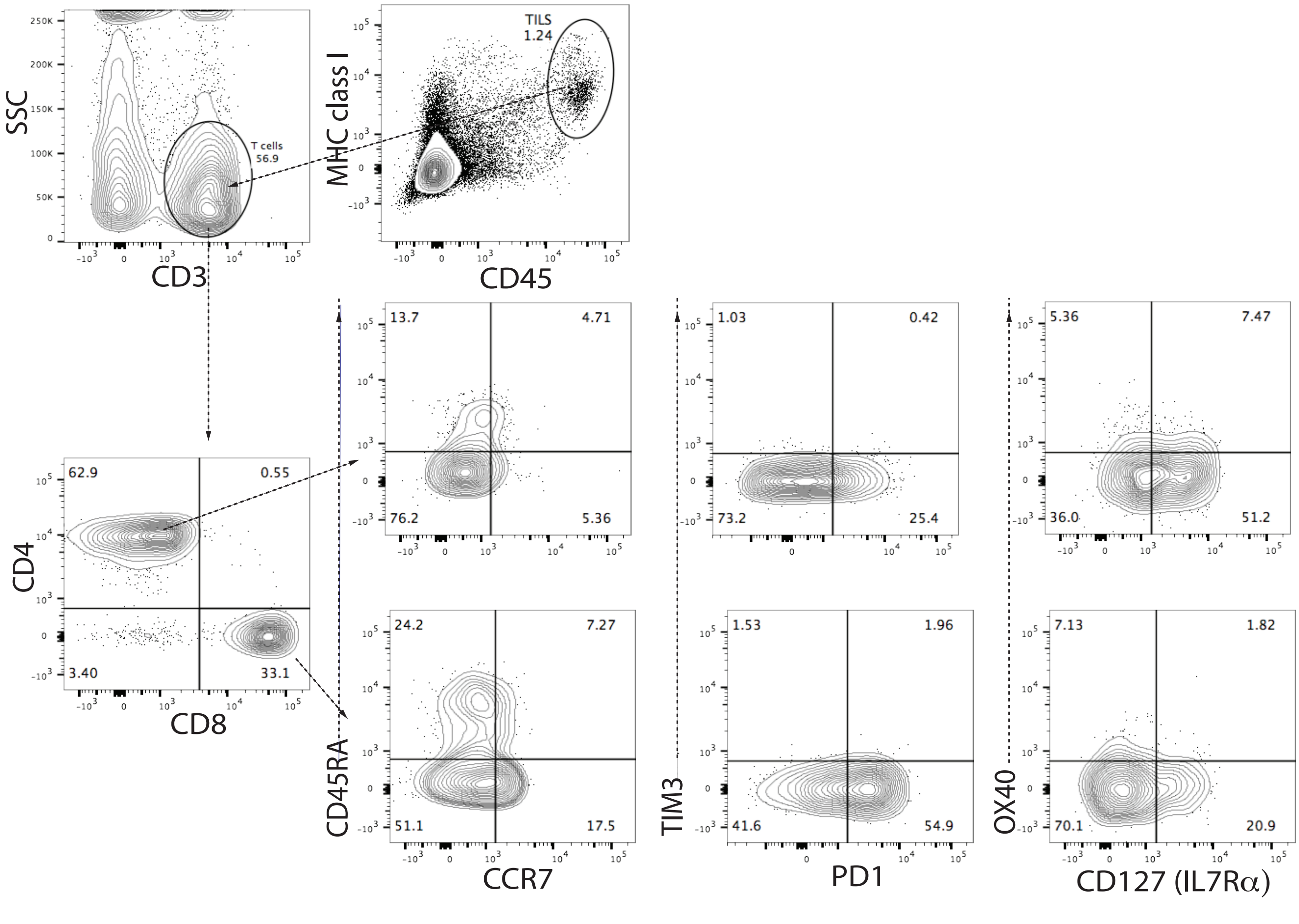

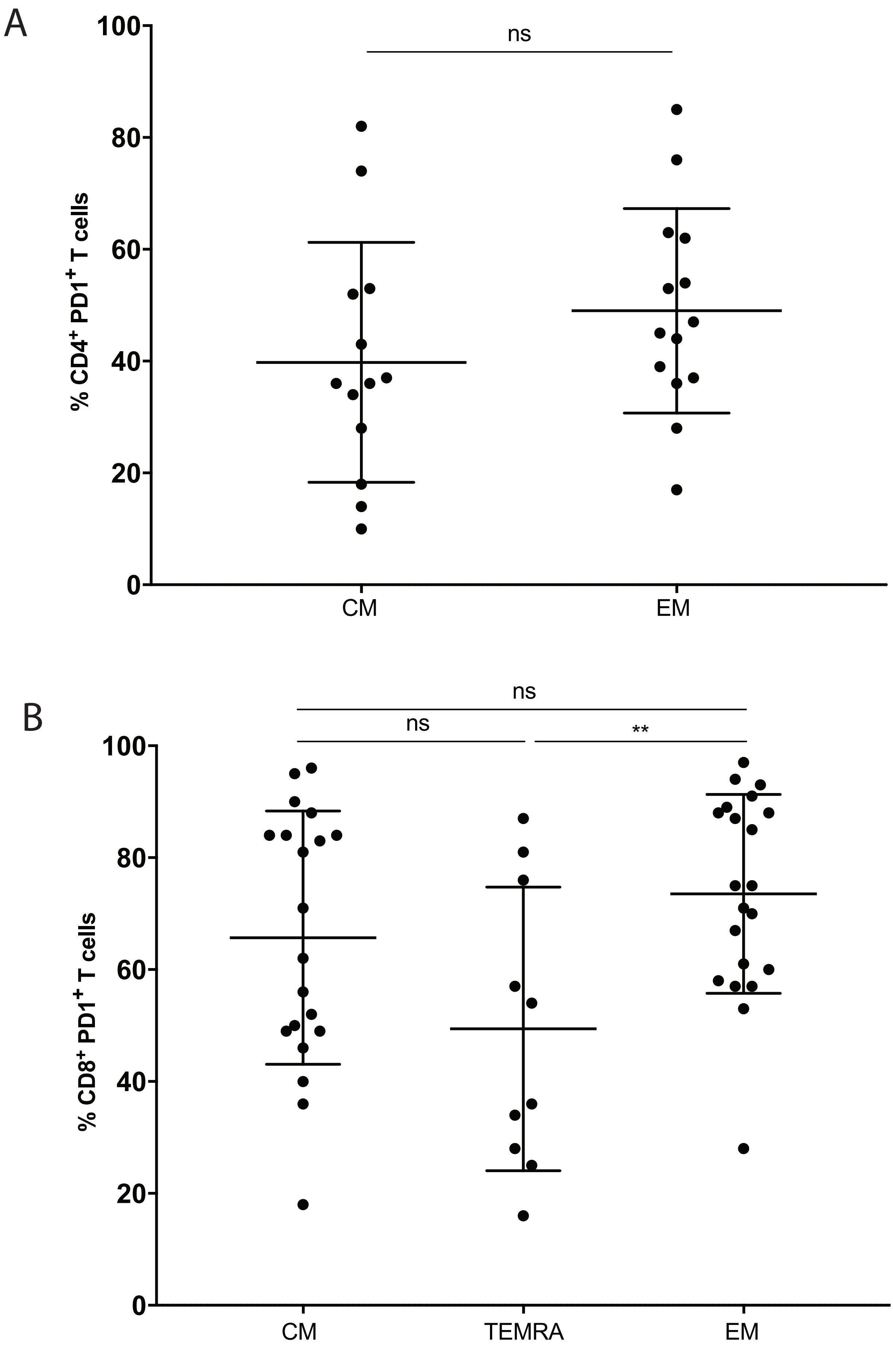

Supplementary Figure 7

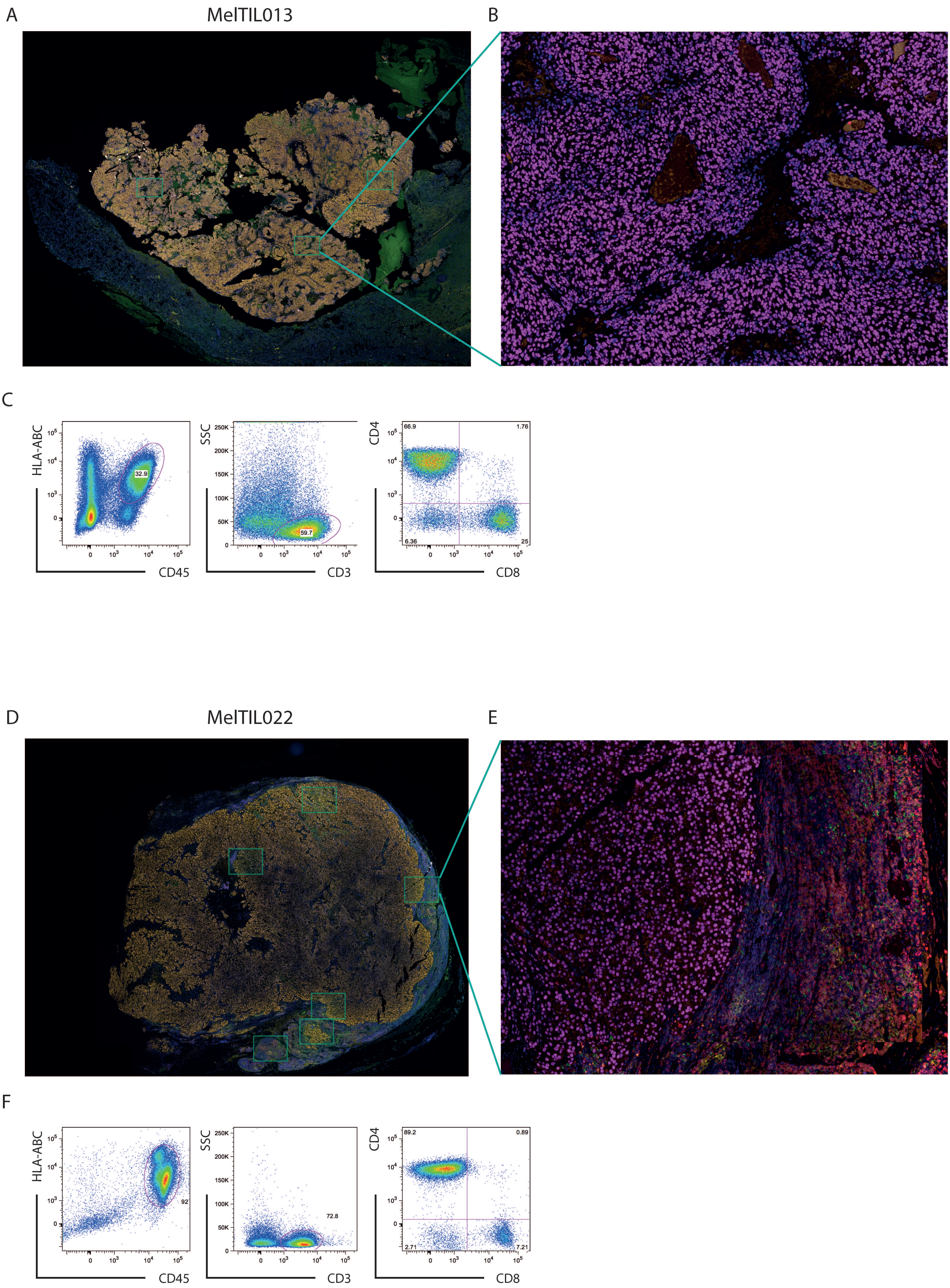

Supplementary Figure 8

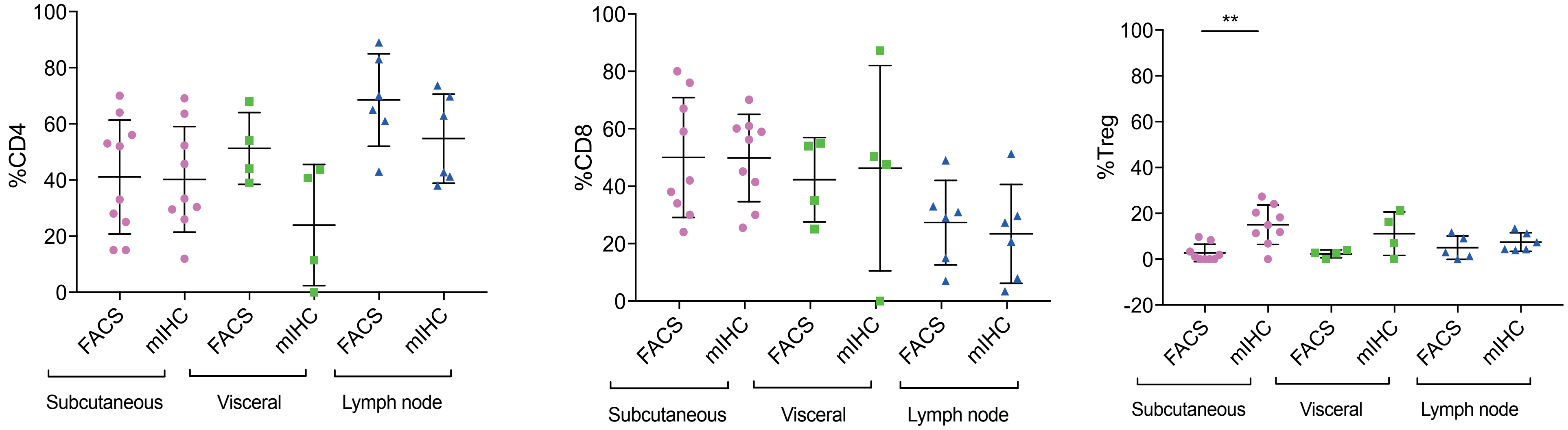

Supplementary Figure 9

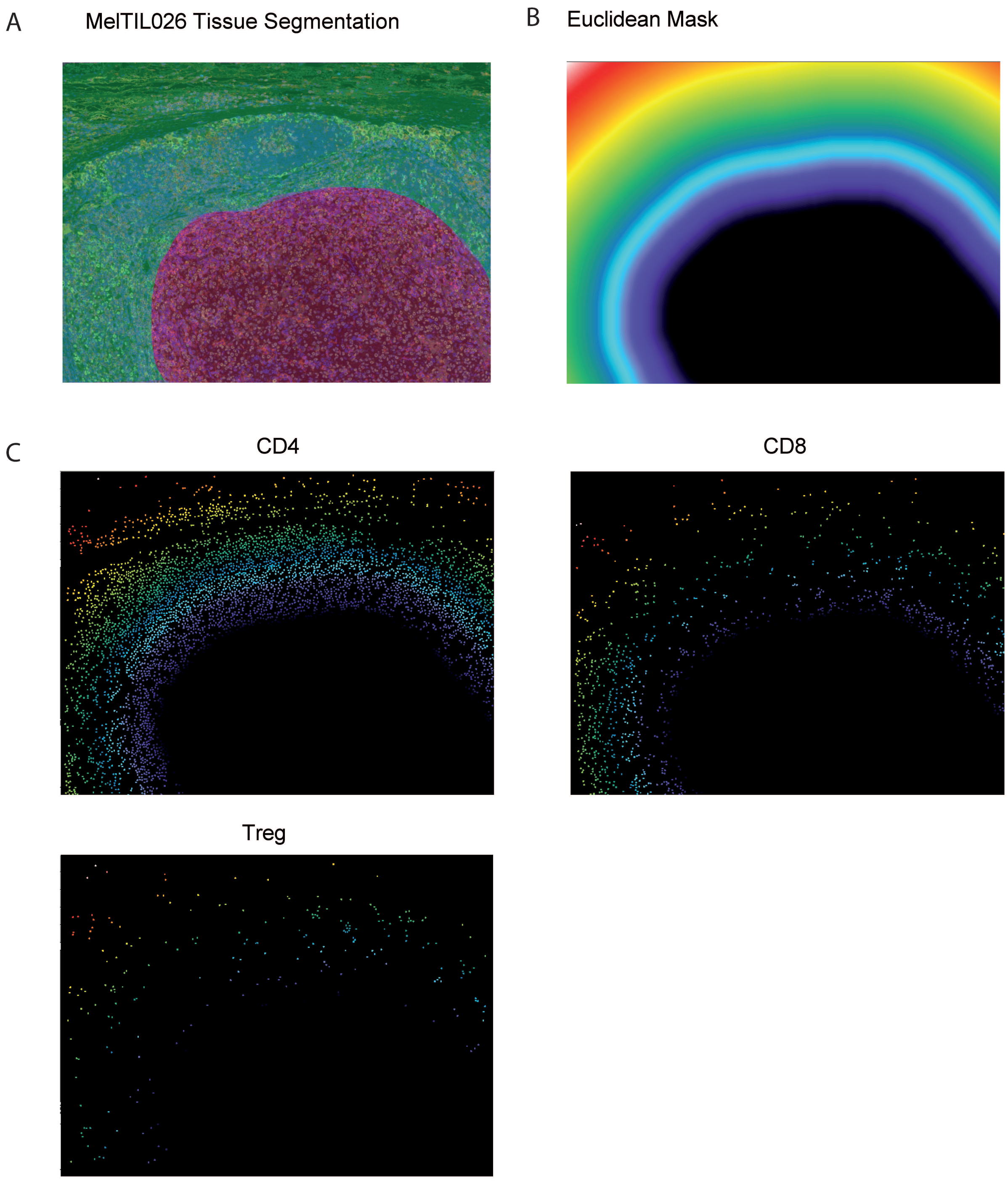

Supplementary Figure 10 - Distance calculation measurements using Metamorph
