# Supplementary material for: Multiplex immunohistochemistry accurately defines the immune context of metastatic melanoma
Source: Sci Rep. 2018 Jul 24;8:11158. doi: 10.1038/s41598-018-28944-3 (PMC6057961; doi:10.1038/s41598-018-28944-3)
Supplement: Supplementary file 2 — Supplementary Tables [file 41598_2018_28944_MOESM2_ESM.docx]

**­­­­Multiplex immunohistochemistry accurately defines the immune context of metastatic melanoma**

Halse H^1#^, Colebatch AJ^2#^, Petrone P^1^, Henderson MA^1^, Mills JK ^1,3^, Snow H^3^, Westwood JA^1^, Sandhu S^2,4^, Raleigh JM^2^, Behren A^5,7^, Cebon J^5,7^, Darcy PK^1,4^, Kershaw MH^1,4^, McArthur GA^2^, Gyorki DE^1,3,6*^ and Neeson PJ^1,4*^.

Affiliations:

(1) Cancer Immunology Research (2) Division of Cancer Medicine Melanoma Program (3) Division of Cancer Surgery

Peter MacCallum Cancer Centre, 305 Grattan Street, Melbourne, 3000.

(4) Sir Peter MacCallum Department of Oncology, The University of Melbourne, Parkville, Victoria, 3052, Australia.

(5) Olivia Newton John Cancer Research Institute, Heidelberg, Victoria, 3084, Australia.

(6) Department of Surgery, University of Melbourne, Parkville, Victoria, 3052, Australia.

(7) School of Cancer Medicine, La Trobe University, Bundoora, 3086, Australia

* denotes equal senior author

# denotes equal first author

Corresponding author: paul.neeson@petermac.org

**Supplementary Table 1**. Demographic features of patients enrolled in study.

|  | Total |
| --- | --- |
| Number | 21 |
| Median age (range) | 70 (38-94) |
| Median time from diagnosis to metastasectomy (range) | 46 (0-470) |
| prior metastasectomy | 9 |
|  |  |
| **Disease stage** |  |
| III | 8 (38%) |
| IV | 13 (62%) |
|  |  |
| **Site of metastasectomy** |  |
| cutaneous/subcutaneous metastasis | 10 (48%) |
| nodal metastasis | 7 (32%) |
| lung metastasis | 2 (10%) |
| visceral metastasis | 2 (10%) |
|  |  |
| **Prior immunotherapy** | 4 (19%) |

**Supplementary Table 2. Metastatic melanoma TIL distribution.**

Shown are data for individual patient metastatic melanoma including tissue site, IT TILs were assessed by the TCGA scoring system by mIHC (using the T cell OPAL panel), and by a pathologist on a H&E section. For both mIHC and pathologist IT TIL assessment, the TIL data was scored for distribution, density and overall score. Also listed are individual biopsy TIL% (IT and average per high powered field) via mIHC or FACS, plus %CD3 by FACS and mIHC.

**Supplementary Table 3. Metastatic melanoma cohort biopsy immune context and patient checkpoint inhibitor response data.**

Shown are data for individual patient metastatic melanoma including tissue site, phenotype, checkpoint inhibitor (CBI) therapy prior to, or after surgery and the clinical response. Also shown is the biopsy IT TIL score, TIL description, PDL1^+^ melanoma or macrophages and the immune context category. Abbreviations include ipi (Ipilimumab), pembro (Pembrolizumab), nivo (nivolumab). Clinical response (Immune-related response criteria ([1](#_ENREF_1))) is listed as CR (complete response), PR (partial response), SD (stable disease), PD (progressive disease).

1. Wolchok JD*, et al.* (2009) Guidelines for the evaluation of immune therapy activity in solid tumors: immune-related response criteria. *Clin Cancer Res* 15(23):7412-7420.
